# Pdcd10-Stk24/25 complex controls kidney water reabsorption by regulating Aqp2 membrane targeting

**DOI:** 10.1172/jci.insight.142838

**Published:** 2021-06-22

**Authors:** Rui Wang, Shi-Ting Wu, Xi Yang, Yude Qian, Jaesung P. Choi, Rui Gao, Siliang Song, Yixuan Wang, Tao Zhuang, Justin J.L. Wong, Yuzhen Zhang, Zhiming Han, Hua A. Lu, Stephen I. Alexander, Renjing Liu, Yin Xia, Xiangjian Zheng

**Affiliations:** 1 Department of Pharmacology, School of Basic Medical Sciences, Tianjin Medical University, China.; 2 Lab of Cardiovascular Signaling, Centenary Institute, and Sydney Medical School, University of Sydney, Sydney, Australia.; 3 Key Laboratory of Arrhythmias of the Ministry of Education of China, Research Center for Translational Medicine, Shanghai East Hospital, Tongji University School of Medicine, Shanghai, China.; 4 Epigenetics and RNA Biology Program Centenary Institute and Sydney Medical School, University of Sydney, Sydney, Australia.; 5 State Key Laboratory of Stem Cell and Reproductive Biology, Institute of Zoology, Chinese Academy of Sciences, Beijing, China.; 6 Division of Nephrology, Department of Medicine, Massachusetts General Hospital and Harvard Medical School, Boston, Massachusetts, USA.; 7 Department of Pediatric Nephrology, The Children’s Hospital at Westmead and Centre for Kidney Research, Sydney Medical School, University of Sydney, Sydney, Australia.; 8 Vascular Epigenetics Laboratory, Victor Chang Cardiac Research Institute, Sydney, Australia.; 9 School of Biomedical Sciences, Faculty of Medicine, The Chinese University of Hong Kong, Hong Kong, China.

**Keywords:** Nephrology, Epithelial transport of ions and water, Mouse models

## Abstract

*PDCD10*, also known as *CCM3*, is a gene found to be associated with the human disease cerebral cavernous malformations (CCMs). PDCD10 forms a complex with GCKIII kinases including STK24, STK25, and MST4. Studies in *C*. *elegans* and *Drosophila* have shown a pivotal role of the PDCD10-GCKIII complex in maintaining epithelial integrity. Here, we found that mice deficient of *Pdcd10* or *Stk24/25* in the kidney tubules developed polyuria and displayed increased water consumption. Although the expression levels of aquaporin genes were not decreased, the levels of total and phosphorylated aquaporin 2 (Aqp2) protein in the apical membrane of tubular epithelial cells were decreased in *Pdcd10*- and *Stk24/25*-deficient mice. This loss of Aqp2 was associated with increased expression and membrane targeting of Ezrin and phosphorylated Ezrin, Radixin, Moesin (p-ERM) proteins and impaired intracellular vesicle trafficking. Treatment with Erlotinib, a tyrosine kinase inhibitor promoting exocytosis and inhibiting endocytosis, normalized the expression level and membrane abundance of Aqp2 protein, and partially rescued the water reabsorption defect observed in the Pdcd10-deficient mice. Our current study identified the PDCD10-STK-ERM signaling pathway as a potentially novel pathway required for water balance control by regulating vesicle trafficking and protein abundance of AQP2 in the kidneys.

## Introduction

Water homeostasis is critical for all physiological processes, and water channel–mediated reabsorption by the kidney is essential to maintain the body’s water balance. The kidneys express numerous aquaporins (AQP) where AQP1–4 and AQP7 are the major AQP involved in water handling. AQP1 and AQP7 are mainly expressed in the proximal tubules, while AQP2–4 are expressed in the connecting tubules and collecting ducts (1–3). Functional AQP2 localizes in the apical membrane of epithelial cells to transport water from lumen to cells, while AQP3 and AQP4 are found in the basolateral membrane of epithelial cells to transport water from cells to the interstitium (1, 2, 4, 5). The localization of AQP2 on the luminal membrane is a key regulatory node for water absorption by the kidney tubules. The hypothalamus senses osmolality to secrete arginine vasopressin (AVP), and AVP interacts with vasopressin V2 receptor (V2R) in kidney epithelial cells to stimulate AQP2 phosphorylation and promote its translocation to the luminal membrane (4). The AVP-V2R-AQP2 axis is the major regulatory pathway for water reabsorption in the mammalian body.

Mutations in V2R and AQP2 genes or drug-induced insufficient response to AVP cause nephrogenic diabetes insipidus (NDI), a water balance disorder that is characterized by polydipsia, polyuria and hypotonic urine (6–8). A large body of studies supports cAMP-mediated PKA activation as the key signaling connection between AVP and AQP2 regulation, including gene expression, posttranslational modification, and membrane targeting. Mounting evidence now suggests that other signaling pathways such as PGE2, cGMP, and calcium signaling can control both AVP-dependent and -independent regulation of AQP2 membrane trafficking and water reabsorption (6, 9–14). Given that specific and effective treatments for NDI are still lacking, the identification of alternative mechanisms regulating AQP2 membrane expression and trafficking, as well as water handling, may lead to novel therapeutic options.

*PDCD10* is a gene associated with cerebral cavernous malformation. The *PDCD10* gene encodes the highly conserved PDCD10 adaptor protein that forms a protein complex with other cerebral cavernous malformation (CCM) disease–associated proteins, KRIT1 and CCM2, in endothelial cells to maintain vessel integrity (15, 16). In addition to the KRIT1-CCM2-PDCD10 complex, PDCD10 is also a component of the STRIPAK complex and interacts with striatin and germinal center kinases (GCK) subfamily of sterile-20 like kinases (STKs), including STK24, STK25, and MST4 (16, 17). Loss of PDCD10 specifically in endothelial cells leads to severe vascular phenotypes, including defective vessel lumen formation in embryos and CCM lesion formation in postnatal mice (18, 19). PDCD10 is also expressed in epithelial cells. In invertebrates, deletion of *Pdcd10* leads to seamless tube dilation in the trachea of *Drosophila* and cyst formation in the excretory tube of *C*. *elegans* (20, 21). In mammals, PDCD10 has been shown to be essential to maintain gut barrier function, and a defective gut barrier in the *Pdcd10*-deficient mice contributes to more aggressive CCM lesion formation (22). The role of PDCD10 in other mammalian epithelial systems has not been explored.

In this study, we have identified PDCD10-STK signaling as a regulatory pathway to control the protein abundance and membrane distribution of AQP2 in kidney tubular epithelial cells, thus serving as a previously unidentified mechanism for body water balance regulation.

## Results

### Renal tubule–specific deletion of Pdcd10 impairs urine concentration and increases water intake.

Cdh16, also known as kidney specific protein (Ksp), is expressed in kidney distal nephrons including the loop of Henle, distal convoluted tubules, and collecting ducts (23). We crossed the *Cdh16-Cre* mice (24) with the *Rosa^YFP^* reporter mice (25) to determine whether the *Cdh16-Cre* can drive efficient recombination in the kidneys, and we observed strong YFP expression in the developing distal tubules and collecting ducts of neonatal kidneys (Supplemental Figure 1, A and B; supplemental material available online with this article; https://doi.org/10.1172/jci.insight.142838DS1) and adult kidneys (Supplemental Figure 1, C and D). Single-cell sequencing data of human kidneys (26) also showed that *CDH16* is specifically expressed in collecting duct cells and loop of Henle (Supplemental Figure 1, E and F). To investigate the specific role of Pdcd10 in kidney epithelial cells, we crossed the *Pdcd10^fl/fl^* mice with the *Cdh16-Cre* transgenic mice to generate the *Cdh16-Cre;Pdcd10^fl/fl^* line (denoted hereafter as the *Pdcd10^KspKO^* mice). These mice lack Pdcd10 specifically in the kidney distal tubules and collecting ducts. Quantitative PCR (qPCR) analysis and Western blotting confirmed a significant reduction in *Pdcd10* expression in the kidney tubules of the *Pdcd10^KspKO^* mice compared with littermate controls (Supplemental Figure 2, A and B). No differences in body weight were observed between *Pdcd10^KspKO^* and littermate control mice from 4 weeks to 20 weeks of age (Supplemental Figure 2, C and D). There was no evidence of gross morphological differences between the control and KO kidneys (Supplemental Figure 2E). H&E staining (Supplemental Figure 2, F and G) and Sirius Red staining (Supplemental Figure 2, H and I) of histological sections of kidneys at weaning (P21) also did not reveal any obvious structural anomalies or fibrosis in the *Pdcd10^KspKO^* mice.

**Figure 1:**
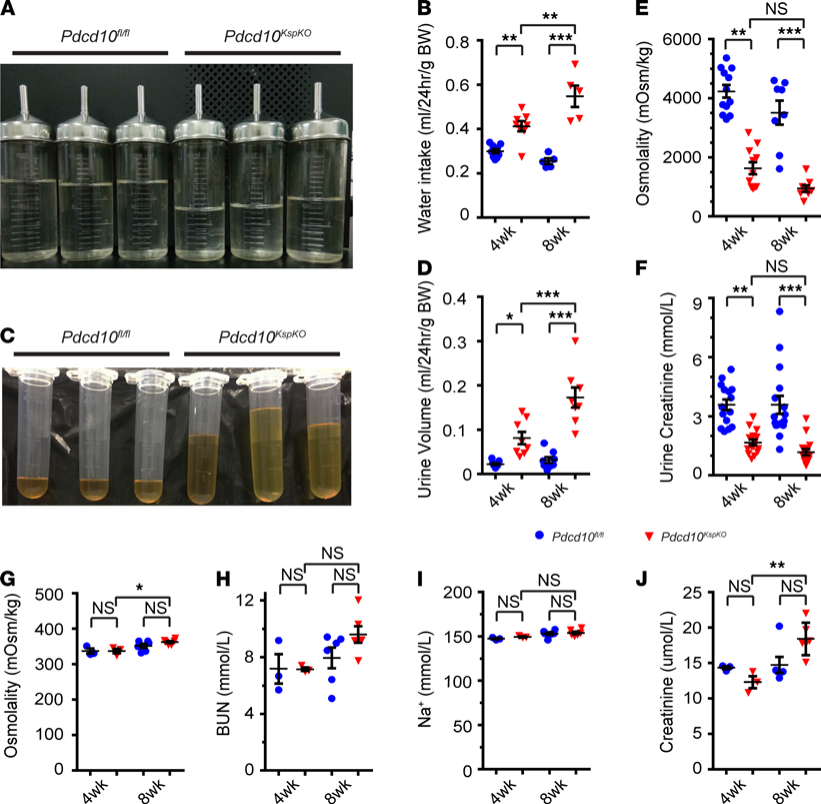
Loss of *Pdcd10* causes polyuria with low urine osmolality. (**A**–**D**) Images and quantitative plots show that *Pdcd10^KspKO^* mice consume more water (**A** and **B**) and excrete more urine (**C** and **D**) than littermate control mice (*n* = 8–9 for 4-week-old [4wk] mice, *n* = 5–8 for 8wk mice). (**E** and **F**) Quantitative plots show the decreased urine osmolality (**E**) and urine creatinine concentration (**F**) of *Pdcd10^KspKO^* mice in comparison with littermate controls at 4 and 8 weeks of age (*n* = 11–15 for 4wk mice, *n* = 8–16 for 8wk mice). (**G**–**J**) Quantitative plots show no significant difference of plasma osmolality (**G**), blood urea nitrogen (**H**), serum concentration of sodium (**I**), and creatinine (**J**) between control and *Pdcd10^KspKO^* mice at 4 and 8 weeks of age, with the exception of a mild increase of serum creatinine (**J**) in *Pdcd10^KspKO^* mice at 8 weeks of age (*n* = 3 for 4wk mice, *n* = 6 for 8wk mice). Data are presented as mean ± SEM. Asterisks indicate significance relative to the control group determined by 1-way ANOVA. *P < 0.05; **P < 0.01; ***P < 0.001.

Functional renal analyses revealed that the *Pdcd10^KspKO^* mice consumed more water (Figure 1, A and B) and excreted more urine (Figure 1, C and D) compared with littermate controls. The urine osmolality was decreased in the KO mice (Figure 1E), and concentration of metabolites and ions in the urine — including creatinine (Figure 1F), urea, uric acid, Na^+^, K^+^, and Cl^–^ (Supplemental Figure 3A) — were all significantly reduced in the *Pdcd10^KspKO^* mice compared with that of the littermates. There were no significant differences in serum osmolality (Figure 1G), blood volume, or blood pressure (Supplemental Figure 3B) between the *Pdcd10^KspKO^* mice and the littermates of the same age. The levels of blood urea nitrogen (BUN) (Figure 1H) — and concentrations of Na^+^ (Figure 1I), uric acid, K^+^, Cl^–^, Ca^2+^, and PO_4_^–^ (Supplemental Figure 3C) — in serum were also not significantly altered between the groups of same age. The serum concentration of creatinine was not altered at 4 weeks of age, but it slightly increased by 8 weeks of age in the *Pdcd10^KspKO^* mice (Figure 1J). In summary, these data suggest that loss of *Pdcd10* specifically in the tubular epithelial cells causes defects in water reabsorption and produces diluted urine with no significant effects on water-electrolyte balance.

In endothelial cells, PDCD10 can form a single protein complex with KRIT1 and CCM2 to maintain blood vessel integrity (27). To determine whether KRIT1 and CCM2 also play roles in regulating kidney epithelial cell function, or whether this function is unique to PDCD10, we generated the *Cdh16-Cre;Krit1^fl/fl^* (denoted *Krit1^KspKO^*) and *Cdh16-Cre;Ccm2^fl/fl^* (denoted *Ccm2^KspKO^*) mice. Water consumption, urine production, and urine osmolality were not altered between these *Krit1-* or *Ccm2*-deficient mice and their littermate controls (Supplemental Figure 4). These results indicate that the regulation of body water balance is not a general function of CCM complex proteins — rather, this function is unique to Pdcd10.

### Pdcd10 deficiency decreases Aqp2 protein abundance and alters membrane localization.

**Figure 2:**
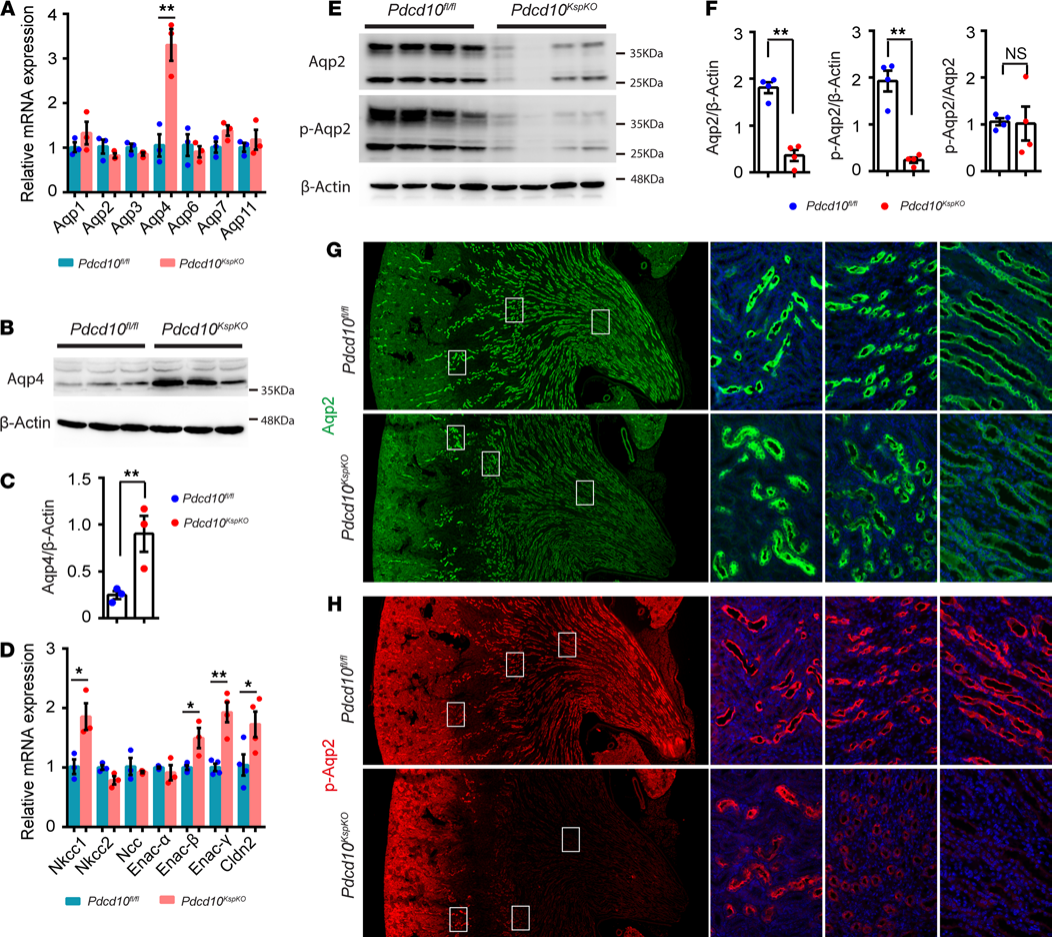
Deletion of *Pdcd10* decreases Aqp2 and pS256-Aqp2 protein levels in the kidney medulla. (**A**) Relative expression level of aquaporin genes in the kidney of control and *Pdcd10*-deficient mice at 4 weeks of age (*n* = 3 for each group). (**B** and **C**) Western blots and quantification of Aqp4 protein levels in the whole kidney lysates of control and *Pdcd10^KspKO^* mice at 4 weeks of age (*n* = 3 for each group). (**D**) Relative expression level of ion channel genes in the kidney of control and *Pdcd10*-deficient mice at 4 weeks of age (*n* = 3–4 for each group). (**E** and **F**) Representative Western blots and quantification of Aqp2 and pS256-Aqp2 protein in the whole kidney lysates of control and *Pdcd10*-deficient mice at 4 weeks of age (*n* = 4 for each group). The lower and upper bands on blots probed with anti-Aqp2 and anti–pS256-Aqp2 represent nonglycosylated and glycosylated forms of Aqp2 or pS256-Aqp2, respectively. (**G** and **H**) Immunofluorescence staining of Aqp2 (green, **G**) and pS256-Aqp2 (red, **H**) on kidney sections of control and *Pdcd10^KspKO^* mice at 4 weeks of age. Boxed regions in the left panels (original magnification, x40) represent areas of junction regions of cortex and medulla, outer medulla, and inner medulla and are shown at higher magnification (original magnification, x400) in the 3 panels to the right, from left to right respectively. Data in the quantitative plots are presented as mean ± SEM, and significance was determined using unpaired *t* test. **P < 0.01; *P < 0.05.

Active transport of water via the AQP channels is essential for water reabsorption and maintaining urine concentration. To determine if the water reabsorption defects in the *Pdcd10^KspKO^* mice were the result of altered expression of the AQP, we performed qPCR analysis using total RNA isolated from whole kidneys. The expressions of AQP were largely unchanged between the *Pdcd10^KspKO^* mice and littermate controls, with the exception of *Aqp4*, which was increased by greater than 3-fold in *Pdcd10^KspKO^* mice (Figure 2A). The increased expression of Aqp4 at the protein level was further confirmed by Western blotting analysis (Figure 2, B and C). In addition to AQP, we also assessed the expression levels of other transporter genes in collecting ducts, distal tubules, and thick ascending limb. Gene expression for the β and γ subunits of the epithelial sodium channel ENaC and the sodium-potassium-chloride cotransporter NKCC1 were all significantly increased in the *Pdcd10^KspKO^* mice compared with controls (Figure 2D). The increased expression of these genes is probably a compensatory response to increase the efficiency of sodium reabsorption from more diluted urine passing through collecting ducts.

AQP4 is a basolateral water channel expressed in the kidney collecting ducts, and it transports water from tubular cells to the interstitium. Therefore, an increase in the expression of Aqp4 observed in the *Pdcd10^KspKO^* mice (Figure 2, A–C) was not consistent with the impaired water reabsorption phenotype seen in the KO mice (Figure 1). We hypothesized that this upregulation may be a compensatory response to the impaired function of other AQP proteins, namely Aqp2, which has an established role in regulating body-water homeostasis through regulation of collecting duct permeability. While the qPCR results demonstrated a nonsignificant reduction in *Aqp2* mRNA expression in the *Pdcd10^KspKO^* mouse kidneys (Figure 2A), Western blotting analysis, on the other hand, revealed striking downregulation in both total Aqp2 and phosphorylated Aqp2 (pS256-Aqp2) in the *Pdcd10^KspKO^* kidneys (Figure 2E); the ratio of pS256-Aqp2/Aqp2 remains the same between control and *Pdcd10^KspKO^* kidneys (Figure 2F).

Immunostaining of Aqp2 and pS256-Aqp2 further localized the decreased expression to the renal medulla and papilla in the *Pdcd10*-deficient kidneys (Supplemental Figure 5). The expression of both Aqp2 and pS256-Aqp2 decreased progressively from the cortex to the inner medulla of the kidneys (Figure 2, G and H). The decrease in pS256-Aqp2 was more pronounced than that of total Aqp2 (Supplemental Figure 5 and Figure 2, G and H) such that the expression of pS256-Aqp2 in the medulla and papilla of the *Pdcd10^KspKO^* kidneys dropped to below detectable levels (Figure 2H).

**Figure 3:**
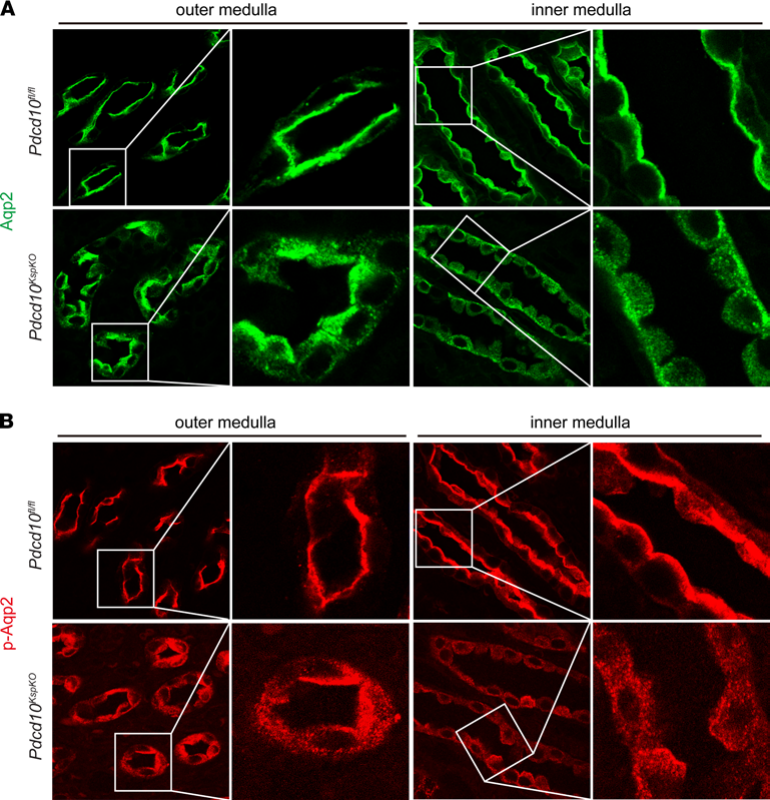
Aqp2 membrane targeting is disrupted in *Pdcd10*-deficient tubular cells. (**A**) Immunofluorescence staining of Aqp2 (green) in renal tubules of outer and inner medulla of control mice and *Pdcd10*-deficient mice. Higher magnification of the selected areas are shown on the right. (**B**) Immunofluorescence staining of pS256-Aqp2 (red) in the renal tubules of the outer and inner medulla of littermate controls and the *Pdcd10*-deficient mice. Higher magnification views of the indicated areas are shown on the right. The images are representatives of 3 repeats of sections from kidneys of mice at 4 weeks of age. Original magnification, ×630 (**A** and **B**).

To determine whether the loss of Pdcd10 affected the subcellular localization of the Aqp2, kidney sections were immunostained with Aqp2 or pS256-Aqp2 and imaged using high-resolution confocal microscopy. In WT littermates, Aqp2 expression was sharply concentrated in the apical plasma membrane of tubular cells in the outer and inner medulla of the kidneys (Figure 3A). In kidney sections of the *Pdcd10^KspKO^* mice, however, Aqp2 was observed to be diffusely distributed in tubular cells (Figure 3A). The expression level and pattern of pS256-Aqp2 mirrored those of Aqp2 (Figure 3B). These results suggest that loss of *Pdcd10* in kidney epithelial cells impair both the distribution and abundance of Aqp2 and p-Aqp2 in kidney collecting duct cells. This may account for the water reabsorption defects observed in the *Pdcd10^KspKO^* mice.

### Pdcd10^KspKO^ mice remain responsive to AVP stimulation and water deprivation.

**Figure 4:**
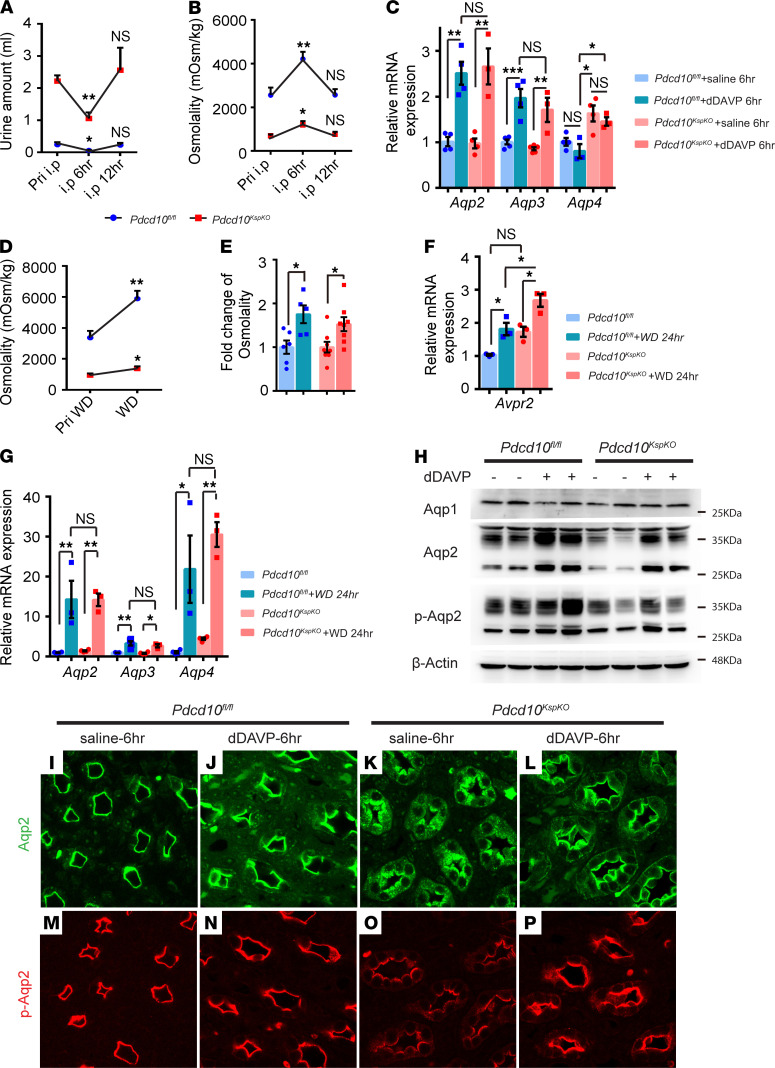
*Pdcd10*-deficient mice maintain proper physiological responses to dDAVP stimulation and water deprivation (WD). (**A** and **B**) Quantitative plots of urine volume (**A**) and osmolality (**B**) before and after administration of dDAVP (i.p. 1 ng/g) to control and *Pdcd10*-deficient mice (*n* = 5 for each group). (**C**) Relative expression level of *Aqp2*, *Aqp3*, and *Aqp4* genes in the kidney of control and *Pdcd10*-deficient mice 6 hours after administration with saline or dDAVP (1 ng/g) (*Pdcd10^fl/fl^* + saline, *n* = 6; *Pdcd10^fl/fl^* + dDAVP, *n* = 3–4; *Pdcd10*^KspKO^ + saline, *n* = 4; *Pdcd10*^KspKO^ + dDAVP, *n* = 3). (**D**) Quantitative plots of urine osmolality in control and *Pdcd10*-deficient mice 24 hours after water deprivation (*Pdcd10^fl/fl^*, *n* = 5–6; *Pdcd10*^KspKO^, *n* = 8). (**E**) The fold change of osmolality in control and *Pdcd10*-deficient mice 24 hours before and after water deprivation (*Pdcd10^fl/fl^*, *n* = 6; *Pdcd10^fl/fl^* + WD, *n* = 5; *Pdcd10*^KspKO,^ *n* = 8; *Pdcd10*^KspKO^ + WD, *n* = 8). (**F**) Relative expression level of *Avpr2* gene in the kidney of control and *Pdcd10*-deficient mice before and 24 hours after water deprivation (*n* = 3 for each group) (**G**) Relative expression level of *Aqp2*, *Aqp3*, and *Aqp4* genes in the kidney of control and *Pdcd10*-deficient mice before and 24 hours after water deprivation (*n* = 3–4 for each group). (**H**) Representative Western blots of Aqp1, Aqp2, and pS256-Aqp2 proteins in the whole kidney lysates of control and *Pdcd10^KspKO^* mice after administration of dDAVP or saline for 6 hours (*n* = 3). (**I**–**P**) Immunofluorescence staining of Aqp2 (green) and pS256-Aqp2 (red) on sections of kidney medulla of control and *Pdcd10*-deficient mice after administration of saline or dDAVP for 6 hours. Data are presented as mean ± SEM using unpaired *t* test (**D**) or 1-way ANOVA (**A**–**C**,**E**–**G**). **P < 0.01; *P < 0.05. Original magnification, ×630 (**I**–**P**).

The absorption and excretion of water are tightly regulated processes, with AVP being the central regulatory hormone. To determine whether *Pdcd10*-deficient tubular cells present defective responses to AVP signaling and water balance stress, *Pdcd10^KspKO^* mice and littermates were treated with desmopressin (dDAVP) (a V2R-selective vasopressin analog) or subjected to water deprivation. Treatment with a single injection of dDAVP (1 ng/g body weight) led to an average decrease of 53% in urine volume (from pretreatment 2.22 mL/6 hours to posttreatment 1.05 mL/6 hours) and a 73% increase in osmolality (from 692 to 1199 mOSM/kg) in the *Pdcd10^KspKO^* mice (Figure 4, A and B). These changes were not statistically different from control mice that had an average of a 75% decrease in urine volume (from pretreatment 0.24 mL/6 hours to posttreatment 0.06 mL/6 hours) and a 64% increase in osmolality (from 2559 to 4208 mOSM/kg) with dDAVP treatment. The absolute water excretion, however, remained significantly different between treated control and *Pdcd10^KspKO^* mice (Figure 4, A and B). Both urine volume and osmolality in the control and *Pdcd10^KspKO^* mice returned to pretreatment levels 12 hours after treatment, when dDAVP was metabolized (Figure 4, A and B). *Aqp2* and *Aqp3* expression in kidneys of both control and KO mice increased following dDAVP treatment, and the magnitude of increase were similar between control and *Pdcd10^KspKO^* mice (Figure 4C). In contrast, dDAVP treatment had no effect on *Aqp4* expression in control mice and did not affect the level of increased Aqp4 expression in *Pdcd10^KspKO^* mice (Figure 4C). These results suggest that the AVP signaling pathway remain functional in the absence of Pdcd10.

Response to water balance stress can be tested using water deprivation studies. Control mice subjected to water deprivation for 24 hours did not alter the expression level of *Pdcd10* in their kidneys (Supplemental Figure 6A). Urine osmolality increased 42% (from 966 to 1371 mOsm/kg) in *Pdcd10*-deficient mice and 75% (from 3362 to 5882 mOsm/kg) in littermate controls (Figure 4, D and E). Water deprivation increased the expression level of *Avpr2* in kidneys of both control and *Pdcd10^KspKO^* mice (Figure 4F). The expression level of *Avpr2* was higher in *Pdcd10^KspKO^* mice than in control mice under both untreated and water-deprivation conditions (Figure 4F). The AVP mRNA level in hypothalamus was increased only in *Pdcd10^KspKO^* mice, but not in control mice, when subjected to water deprivation for 24 hours (Supplemental Figure 6B). Similar to dDAVP treatment, water deprivation increased the expression of *Aqp2* and *Aqp3* to similar levels in the control and *Pdcd10^KspKO^* kidneys (Figure 4G). In contrast to dDAVP treatment, water deprivation increased *Aqp4* expression in the control mice, but the level of increase was significantly lower than that induced by *Pdcd10* deficiency. Water deprivation had no additional effect on *Aqp4* expression in the *Pdcd10^KspKO^* kidneys (Figure 4G).

Western blotting analysis further confirmed the increase in total Aqp2 and p-Aqp2 in response to dDAVP treatment in both *Pdcd10^KspKO^* mice and their littermate controls (Figure 4H). The level of Aqp1, a water channel mainly expressed in the proximal tubule and descending limb of loop of Henle, was comparable between control and *Pdcd10^KspKO^* mice and was unaffected with dDAVP treatment (Figure 4H). Immunostained kidney sections showed strong Aqp2 and p-Aqp2 localization to the apical or subapical domain in the collecting duct cells in the control mice (Figure 4, I and M). In contrast, decreased expression and diffused localization of Aqp2 (Figure 4K) and p-Aqp2 (Figure 4O) were observed in the collecting duct cells of the *Pdcd10^KspKO^* kidney. Treatment with dDAVP promoted targeting of Aqp2 and p-Aqp2 to the luminal membrane of tubular cells in both control (Figure 4, J and N) and KO mice (Figure 4, L and P), although diffused cytoplasmic Aqp2 and p-Aqp2 staining was still evident and a small fraction of Aqp2 and p-Aqp2 were targeted to the basolateral membrane in the dDAVP-treated kidneys from the *Pdcd10^KspKO^* mice. Collectively, these data indicate that loss of *Pdcd10* in kidney epithelial cells does not directly interfere with the AVP signaling pathway and that the water reabsorption defect observed in the *Pdcd10^KspKO^* mice was due to an AVP-independent pathway that can regulate AQP2 protein level and membrane abundance.

### Pdcd10 regulates vesicle trafficking in cultured collecting duct cells.

**Figure 5:**
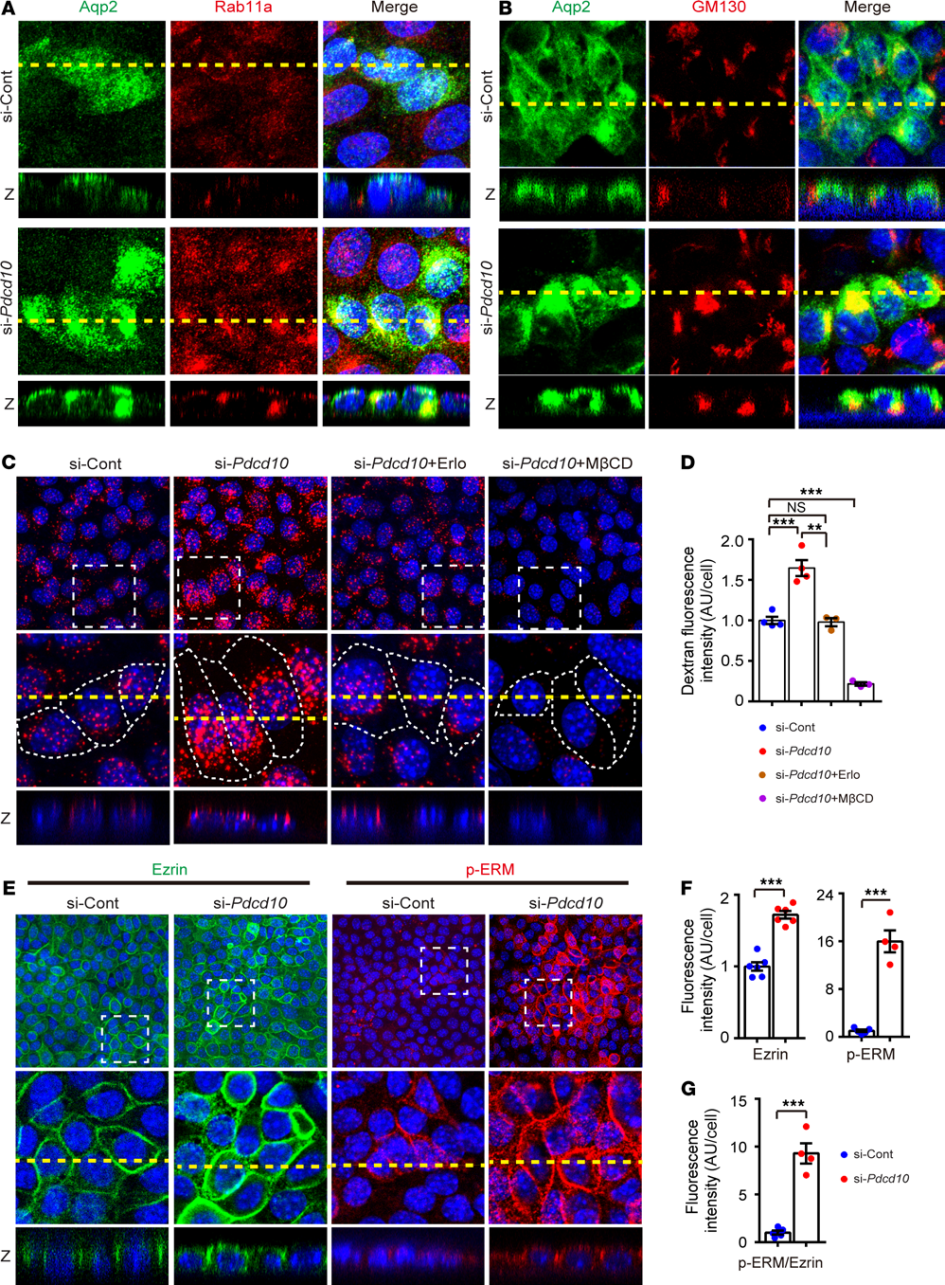
Pdcd10 regulates vesicle trafficking in cultured collecting duct cells. (**A** and **B**) Immunostaining of Aqp2(green) and Rab11a (red) (**A**), and Aqp2(green) and GM130 (red) (**B**), of si-Cont and si-*Pdcd10*–treated mCCD_Cl1_ cells transduced with lentivirus expressing Aqp2. The square panels represent *xy*-sections, and the smaller horizontal strips at the bottom of each square panel represents *z*-sections of reconstructed 3-D confocal stacks at the at the positions indicated by the yellow dashed lines in the *xy* planes. (**C**) Representative images showing the internalization of dextran (red) in si-Cont and si-*Pdcd10*–transfected mCCD_Cl1_ cells, and si-*Pdcd10*–transfected mCCD_Cl1_ cells treated with Erlotinib or methyl-β-cyclodextrin (MβCD). The middle square panels represent higher magnification of the selected areas. The *z*-sections of the cells are indicated by the dashed line at the bottom of each square panel. (**D**) The quantifications of the mean cellular dextran fluorescence intensity; each dot represents fluorescence intensity normalized to si-Cont (si-Cont and si-*Pdcd10*, *n* = 4; si-*Pdcd10* + Erlo and si-*Pdcd10* + MβCD, *n* = 3). (**E**) Immunostaining of Ezrin (green) and p-ERM (red) in the si-Cont and si-*Pdcd10*–treated mCCD_Cl1_ cells. The middle square panels represent higher magnification of the selected areas. The square panels represent *xy*-sections, and the horizontal strips at the bottom of each square panel represent *z*-sections of reconstructed 3-D confocal stacks. (**F**) The quantification of mean cellular Ezrin and p-ERM fluorescence intensity; each dot represents fluorescence intensity normalized to si-Cont (*n* = 6 for each group of Ezrin; si-Cont of p-ERM, *n* = 5; si-*Pdcd10* of p-ERM, *n* = 4). The *n* value represents the field of visions in different experiments. (**G**) The quantification of mean cellular p-ERM fluorescence intensity after normalization with total ezrin abundance. Data are presented as mean ± SEM using unpaired *t* test (**F** and **G**) or 1-way ANOVA (**D**). ***P < 0.001; **P < 0.01. Original magnification, ×630 (**A** and **C**); ×400 (**B** and **E**).

The membrane abundance of Aqp2 is controlled by a balance between membrane targeting and internalization, with the AVP-cAMP-PKA signaling cascade being the major promoter of Aqp2 exocytosis. To test whether Pdcd10 can regulate the vesicle trafficking pathway, we cultured an immortalized mouse collecting duct cell line, mCCD_Cl1_ cells, on transwell membranes to facilitate cell polarity establishment in culture (28). Since the mCCD_Cl1_ cells lack endogenous Aqp2 expression when cultured in dishes, we transduced mCCD_Cl1_ with lentivirus to express Aqp2 and monitor its intracellular vesicle distribution and translocation. Pdcd10 was effectively knocked down using siRNA (Supplemental Figure 7). Immunostaining of Aqp2 and vesicle trafficking regulator Rab11 showed Pdcd10 knockdown increased intracellular Rab11 signal and it colocalization with Aqp2 (Figure 5A). Immunostaining of Aqp2 with the Golgi complex marker GM130 also indicated increased cytosolic localization of Aqp2, as well as more compact and intense staining of the Golgi complexes (Figure 5B). Importantly, the colocalization between GM130 and Aqp2 was increased in the si-*Pdcd10* cells compared with that in the si-Cont cells (Figure 5B). The accumulation of Rab11and GM130 in the intracellular compartment are indicators of impaired vesicle trafficking.

To more specifically assess whether Pdcd10 regulates endocytosis activity, fluorescence dextran uptake assays uptake assays were performed in si-Cont and si-*Pdcd10*–treated mCCD_Cl1_ cells. Markedly increased dextran internalization was observed in the si-*Pdcd10* cells when compared with si-Cont cells (Figure 5C). Erlotinib is an EGFR inhibitor previously shown to increase exocytosis, inhibit endocytosis, and increase membrane AQP2 in kidney cells (29). Treatment with Erlotinib reversed the increased dextran internalization in the si-*Pdcd10* mCCD_Cl1_ cells. Methyl-β-cyclodextrin (MβCD), an endocytosis inhibitor extracting cholesterol from the plasma membrane, was used as the positive control (Figure 5, C and D). These results demonstrated that loss of Pdcd10 can enhance endocytosis in kidney cells.

Ezrin, Radixin, and Moesin (ERM) proteins link the plasma membrane with the cellular cortex (30). Ezrin has been shown to promote Aqp2 endocytosis, and knockdown of Ezrin has been reported to increase Aqp2 expression in the plasma membrane (31). Immunostainings revealed that Ezrin was primarily localized to the plasma membrane, and its staining intensity significantly increased in the si-*Pdcd10* cells (Figure 5, E and F). p-ERM expression was minimally detected in the si-Cont mCCD_C11_ cells and, similar to Ezrin, was localized mainly to the plasma membrane. In contrast, loss of *Pdcd10* led to a significant upregulation of p-ERM expression in cultured kidney cells, as well as an increase in the redistribution of p-ERM from the basolateral to apical membrane (Figure 5, E and F). Quantification of the immunostaining data demonstrate a significant upregulation of p-ERM/Ezrin ratio in the si-*Pdcd10* cells, suggesting that Pdcd10 may regulated ERM phosphorylation (Figure 5G). These results indicate that lack of Pdcd10 in kidney tubular cells upregulates Ezrin expression level, its phosphorylation, and apical membrane distribution, which corresponds with increased membrane targeting of AQP2.

### Pdcd10 negatively regulates ERM protein abundance and membrane targeting in vivo.

**Figure 6:**
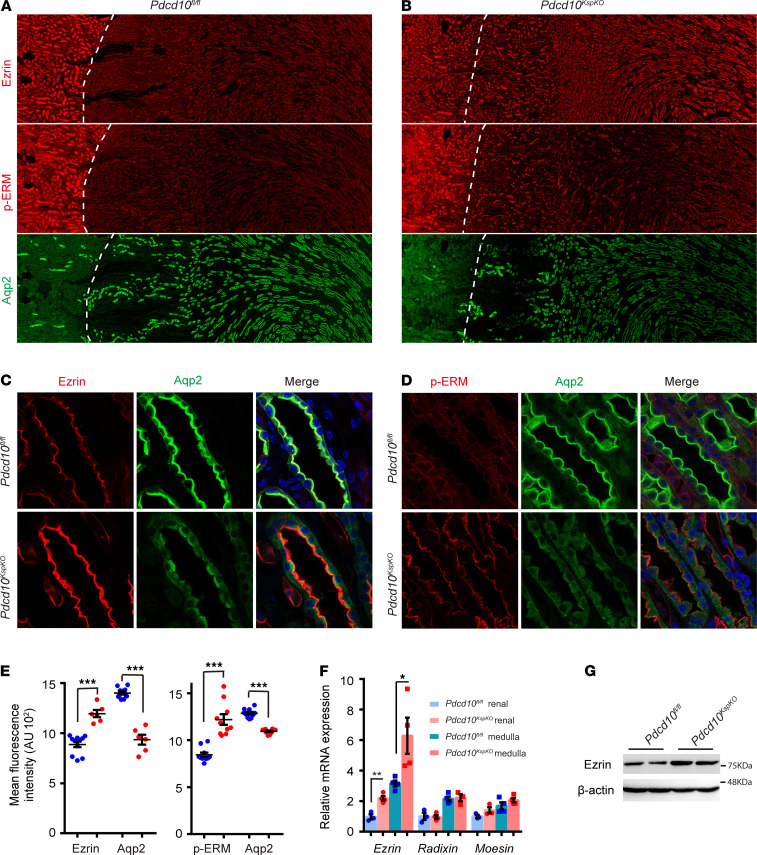
*Pdcd10* deletion increases Ezrin and p-ERM protein abundance and membrane targeting. (**A** and **B**) Immunofluorescence staining of Ezrin and p-ERM demonstrates the protein level of both Ezrin and p-ERM are increased in the medulla of *Pdcd10*-deficient kidneys correlated to the decreased expression of Aqp2 (dashed lines indicate the boundaries between cortex and medulla). (**C** and **D**) High-power confocal images show the increased expression and luminal membrane localization of Ezrin (**C**) and p-ERM (**D**) in tubular cells of the inner medulla of *Pdcd10*-deficient kidneys. Overlay images with Aqp2 staining demonstrate that the increased expression of Ezrin and p-ERM is correlated with the decreased expression and diffused distribution of Aqp2 in tubular cells in *Pdcd10*-deficient mice. The images shown are representative of 3 experiments with kidney samples from mutant and littermate control mice at 4 or 8weeks of age. (**E**) The quantification of mean fluorescence intensity of Ezrin and Aqp2 (left) in medulla (*Pdcd10^fl/fl^*, *n* = 11; *Pdcd10*^KspKO^, *n* = 6), and p-ERM and Aqp2 (right) in medulla (*n* = 10 for each group). (**F**) Relative expression level of *Ezrin*, *Radixin*, and *Moesin* genes in the total kidney and medulla of control and *Pdcd10*-deficient mice at 4 weeks of age (*n* = 3–4 for each group). (**G**) Western blots of Ezrin protein in the renal medulla of control and *Pdcd10^KspKO^* mice at 8 weeks of age. Data are presented as mean ± SEM using unpaired *t* test. ***P < 0.001; *P < 0.05. Original magnification, ×40 (**A** and **B**); ×630 (**C** and **D**).”

To determine whether the upregulation of Ezrin/p-ERM observed in the *Pdcd10* knockdown cells was conserved in vivo, ERM protein expression level was assessed in the kidneys of *Pdcd10^KspKO^* and littermate control mice. In WT mice, both Ezrin and p-ERM were highly expressed in the kidney cortex, with minimal expression in the kidney medulla, while Aqp2 was abundantly expressed in the kidney medulla (Figure 6A). Conversely, in the *Pdcd10^KspKO^* mice, Ezrin and p-ERM were upregulated in the medulla, and Aqp2 expression was downregulated compared with the controls (Figure 6B). Coimmunostaining of Aqp2 with Ezrin or p-ERM further confirmed that expressions of Ezrin (Figure 6C and Supplemental Figure 8A) and p-ERM (Figure 6D and Supplemental Figure 8B) were upregulated in the collecting ducts from the cortex to inner medulla in the *Pdcd10^KspKO^* mice, and this upregulation coincided with significant reduction and diffused expression of Aqp2 (Figure 6, C–E, and Supplemental Figure 8). qPCR analysis revealed that the gene expression of Ezrin, but not Radixin and Moesin, increased in kidney medulla (Figure 6F). The increase of Ezrin protein expression in *Pdcd10^KspKO^* kidney was further confirmed by Western blotting analysis (Figure 6G). Together, these results suggest that Ezrin is a downstream target negatively regulated by Pdcd10 in kidney tubular cells.

### Erlotinib reverses the water reabsorption defect in the Pdcd10^KspKO^ mice.

**Figure 7:**
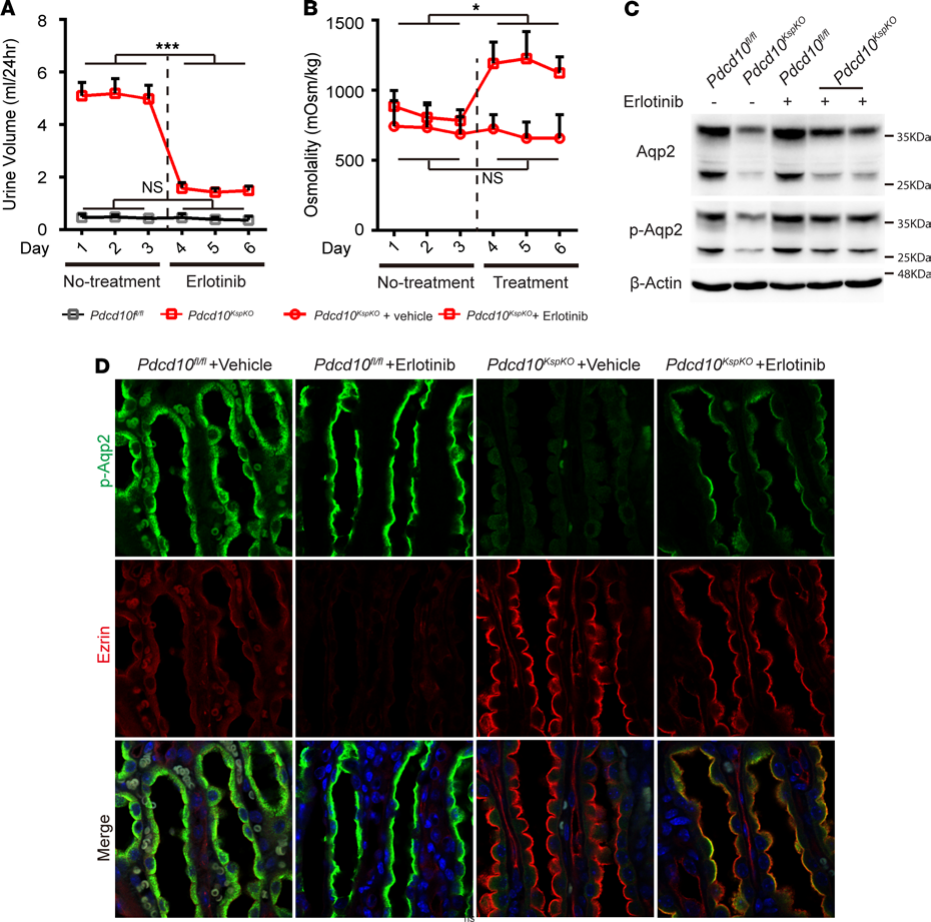
Erlotinib treatment increases urinary concentration and enhances AQP2 abundance in the apical membrane. (**A**) Quantitative plots show decreased urine volume after administration of Erlotinib (100 mg/kg) in *Pdcd10*-deficient mice but not the control mice (*Pdcd10^fl/fl^*, *n* = 4–6; *Pdcd10*^KspKO^, *n* = 5). (**B**) Quantitative plots show increased urine osmolality after administration of Erlotinib, but not vehicle, in *Pdcd10*-deficient mice (*n* = 4–6). (**C**) Representative Western blots show the increase of Aqp2 and pS256-Aqp2 proteins in the whole kidney lysates of *Pdcd10*-deficient mice after administration of Erlotinib for 3 days. (**D**) Immunofluorescence staining of pS256-Aqp2 (green) and Ezrin (red) on sections of kidney medulla of control and *Pdcd10*-deficient mice show the increased p-Aqp2 expression is correlation with the decrease of Ezrin expression after administration of Erlotinib for 3 days. ***P < 0.001; *P < 0.05. Original magnification, ×630 (**D**).

Given that Ezrin and p-ERM levels increased in the *Pdcd10^KspKO^* mice — and Pdcd10 knockdown cells display an increased in endocytosis activity, which was reversible by Erlotinib treatment (Figure 5C) — we reasoned that Pdcd10 may regulate Aqp2 abundance through suppressing Ezrin-mediated endocytosis and that treatment with Erlotinib may be sufficient to reverse the water reabsorption defect observed in *Pdcd10^KspKO^* mice by inhibiting Aqp2 endocytosis. Indeed, Erlotinib treatment (100 mg/kg/day) dramatically decreased urine volume (from average 5.08 to 1.50 mL/day) (Figure 7A) and increased urine osmolarity (from 824.11 to 1179.77 mOSM) (Figure 7B) in the *Pdcd10^KspKO^* mice, but did not significantly alter the urine volume (Figure 7A) and osmolarity (Supplemental Figure 9A) in control mice. Western blotting analysis of kidney protein isolated from control and *Pdcd10^KspKO^* mice treated with vehicle and Erlotinib show increased Aqp2 and p-Aqp2 protein levels following Erlotinib treatment (Figure 7C). Erlotinib treatment promoted p-Aqp2 (Figure 7D) and Aqp2 (Supplemental Figure 9B) accumulation and localization to the apical plasma membrane of epithelial cells in WT kidneys. While p-Aqp2 and Aqp2 were expressed at low levels in the medulla of *Pdcd10^KspKO^* kidney, treatment with Erlotinib significantly increased the expression and accumulation of both p-Aqp2 and Aqp2 in the apical membrane. In contrast, treatment with Erlotinib abolished Ezrin expression in the WT kidneys, and a reduction in Ezrin staining was evident in the Erlotinib-treated *Pdcd10^KspKO^* mice (Figure 7D and Supplemental Figure 9). These in vivo results, together with our cell culture studies (Figure 5), support the notion that loss of *Pdcd10* impairs membrane targeting of Aqp2, leading to a reduction in Aqp2 levels and, consequently, water reabsorption dysfunction in the *Pdcd10^KspKO^* mice. This water reabsorption defect can be partially rescued using Erlotinib to promote membrane targeting of Aqp2.

### Stk24/25 deficiency in renal tubules causes polyuria in vivo.

**Figure 8:**
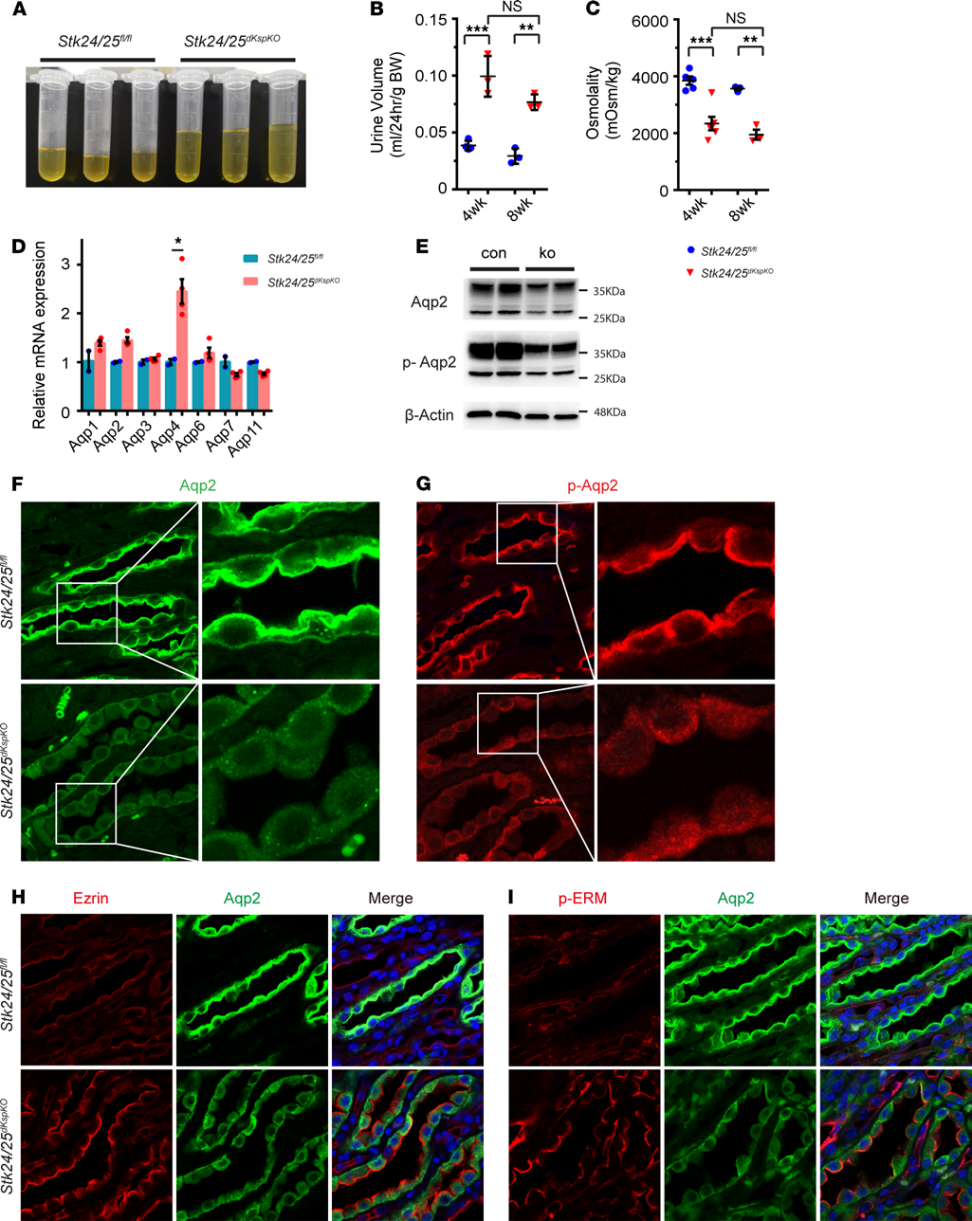
Deletion of *Stk24* and *Stk25*, interacting partners of Pdcd10, recapitulates polyuria phenotype. (**A**) Image shows *Stk24*/*Stk25*-deficient mice excrete more urine than littermate controls at 8 weeks of age. (**B** and **C**) Quantitative plots show increased urine volume (**B**) and decreased urine osmolality (**C**) in *Stk24/Stk25*-deficient mice compared with littermate controls at 4 and 8 weeks of age (*n* = 5–6 for 4wk mice, *n* = 3 for 8wk mice). (**D**) Relative expression level of *Aqp* genes demonstrates no change of expression, except the increase of Aqp4 in *Stk24/Stk25*-deficient kidney (*n* = 3 for each group). (**E**) Representative Western blots show the decreased expression of Aqp2 and pS256-Aqp2 protein in the whole kidney lysates of *Stk24/Stk25*-deficient mice versus control mice at 12 weeks of age. (**F** and **G**) Immunofluorescence staining of Aqp2 (**F**) and pS256-Aqp2 (**G**) on sections of kidney medulla show the deceased expression and diffused distribution of Aqp2 and p-Aqp2 in tubular cells of *Stk24/Stk25*-deficient mice at 8 weeks of age. (**H** and **I**) High-power confocal images show the increased expression and luminal membrane localization of Ezrin (**H**) and p-ERM (**I**) in tubular cells of the inner medulla of *Stk24/Stk25*-deficient kidneys. Overlay images with Aqp2 staining demonstrate that the increased expression of Ezrin and p-ERM is correlated with the decreased expression and diffused distribution of Aqp2 in tubular cells. The images shown are representative of 3 experiments with kidney samples from littermate control and mutant mice at 4 or 8 weeks of age. Data in quantitative plots are presented as mean ± SEM using the unpaired *t* test (**D**) or 1-way ANOVA (**B** and **C**). *P < 0.05, **P < 0.01, ***P < 0.001. Original magnification, ×630 (**F**–**I**).

Pdcd10 directly interacts with Ccm2 to form the Krit1-Ccm2-Pdcd10 protein complex, and mutations in any of these 3 Ccm genes cause Ccm disease. While the *Pdcd10^KspKO^* mice demonstrated a severely defective water reabsorption phenotype (Figure 1), no such defects were evident in the *Krit1^KspKO^* and *Ccm2^KspKO^* mice (Supplemental Figure 4). These data suggest that regulation of renal function is Pdcd10 specific, and the pathway by which Pdcd10 signals through is independent of the canonical Ccm signaling. In addition to Krit1 and Ccm2, Pdcd10 also interacts with the GCKIII subfamily of STKs, including Stk24, Stk25, and Mst4. It is therefore plausible that Pdcd10 cooperate with STKs to regulate Aqp2 membrane targeting and stability, as well as control kidney water reabsorption. Thus, we generated mice that contain the floxed alleles of *Stk24* and *Stk25* (Supplemental Figure 10) and crossed them with the *Cdh16-Cre* mice to produce the *Cdh16-Cre;Stk25^fl/fl^* (*Stk25^KspKO^*) and *Cdh16-Cre;Stk24^fl/fl^;Stk25^fl/fl^* (*Stk24/25^dKspKO^*) mice that lack *Stk25* only or *Stk24* and *Stk25,* respectively, in kidney tubular cells to investigate the role of STKs in the kidneys. No water-handling defect was evident in the *Stk25^KspKO^* mice. Urine volume, urine osmolality, and concentrations of creatinine, Na^+^, K^+^, and Cl^–^ all remained normal in these *Stk25^KspKO^* mice (Supplemental Figure 11, A and B). These results suggest a potential functional redundancy between Stk24 and Stk25. Indeed, the *Stk24/25^dKspKO^* mice excreted more urine (Figure 8, A and B) with lower osmolality (Figure 8C), similar to the phenotype observed in the *Pdcd10^KspKO^* mice. Additionally, ion concentrations in the urine from the *Stk24/25^dKspKO^* mice were also significantly reduced (Supplemental Figure 11C). The serum osmolality and concentration of creatinine, Na^+^, and other common ions in blood were similar between the *Stk24/25^dKspKO^* and control mice (Supplemental Figure 11D). As observed in the *Pdcd10^KspKO^* mice, *Aqp4* expression was elevated in the *Stk24/25^dKspKO^* mice, while the expression of other AQP remained at comparable levels with those of control mice (Figure 8D). Consistent with the role of Pdcd10 in regulating Aqp2 at the posttranslational level, *Stk24/Stk25* deletion reduced Aqp2 and p-Aqp2 protein levels in whole kidney protein lysates (Figure 8E). A dramatic decrease in the expression of Aqp2 and p-Aqp2 was observed in the medulla of *Stk24/25^dKspKO^* kidneys (Figure 8, F and G, and Supplemental Figure 12), with low levels of Aqp2 and pS256-Aqp2 signal detected inside the tubular cells in the *Stk24/25^dKspKO^* kidney and with reduced staining in the apical regions (Figure 8, F and G, and Supplemental Figure 12, B and D).

Consistent with the observations in *Pdcd10^KspKO^* mice, both Ezrin (Figure 8H) and p-ERM (Figure 8I) levels were upregulated in kidney tubular cells of the *Stk24/25^dKspKO^* mice, and this was correlated with the decreased expression and membrane localization of Aqp2 in these cells (Figure 8, H and I, and Supplemental Figure 13). These results suggest that STKs may function together with Pdcd10 to regulate Ezrin and Aqp2. Ezrin serves as a mediator in the Pdcd10-Stk signaling pathway and regulates Aqp2 trafficking and protein abundance in kidney tubular cells. Our data together indicate that Stk24 and Stk25 work simultaneously with Pdcd10 to regulate Aqp2 protein abundance and distribution that, in turn, affect the kidney’s ability to reabsorb water.

## Discussion

Dysfunctional targeting of AQP2 to the epithelial cell membrane is an immediate cause of NDI. Directly promoting AQP2 targeting to the apical membrane can be a potential strategy to treat NDI (6–8, 32). The cAMP-PKA pathway has been reported to be the major signaling pathway that regulate AQP2 membrane targeting; however, no therapeutic treatment based on this pathway has been developed. A few established treatments, such as statin and NSAIDs, were developed with alternative mechanisms to improve membrane localization of AQP2 (1, 7, 9). Here, we identified PDCD10-STK signaling as a negative regulator of ERM protein expression and membrane targeting. Furthermore, the PDCD10-STK complex is necessary to maintain the total AQP2 protein level and sufficient AQP2 expression in the apical membrane of tubular cells to facilitate kidney water reabsorption. A water reabsorption defect is evident in the *Pdcd10^KspKO^* mice, but these mice remain responsive to AVP stimulation and water deprivation stress, suggesting that the regulation of AQP2 expression and membrane targeting by Pdcd10-Stk signaling is independent of the canonical AVP-PKA-cAMP signaling. As a long-term effect, AVP signaling is able to stimulate *Aqp2* and *Aqp3* gene expressions. An absence in the change of *Aqp2* gene expression in the *Pdcd10-* or *Stk24/25*-deficient mice may also suggest that the regulation of PDCD10-STK on AQP2 is accomplished via an AVP-independent mechanism. However, further analysis with blockers for AVP signaling may provide more direct evidence.

Aqp2 and pS256-Aqp2 protein are reduced and diffusely distributed in the tubular cells in both the *Pdcd10^KspKO^* and *Stk24/25^dKspKO^* mice, while *Aqp2* mRNA expression levels remain unchanged. The decrease in Aqp2/pS256-Aqp2 total protein level and apical membrane localization strongly correlate with the significant increase in Ezrin and p-ERM protein levels and their apical membrane localization in kidney tubular cells. It has been reported that increased Ezrin can promote endocytosis and facilitate Aqp2 internalization and degradation in cultured kidney cells (31). In vitro studies have also identified a peptide that dissociates the interaction of Moesin, another member of ERM protein family, from its effectors and promotes Aqp2 translocation to the plasma membrane (33). Our qPCR analyses show Ezrin to be the major form of ERM protein that is expressed in mouse kidneys and regulated by Pdcd10. These data and our in vitro and in vivo studies suggest a posttranslational regulatory mechanism of Aqp2 level by Pdcd10-Stk signaling, where a loss of Pdcd10-Stk promotes the expression and targeting of ERM protein to the apical membrane, which facilitates Aqp2 protein internalization and degradation. This is further supported by the results that Erlotinib can normalize the apical targeting of Aqp2 and rescues the water reabsorption defect in *Pdcd10^KspKO^* mice. Combined, our results strongly suggest Pdcd10-Stk as a potentially novel regulator of Aqp2 protein expression level and membrane targeting. Further studies are necessary to elucidate how Pdcd10-Stk regulates Ezrin expression, intracellular vesicle trafficking, and Aqp2 protein degradation, such as through lysosomal or proteasomal mechanisms.

Both PDCD10 and STK have been shown to be localized in the Golgi apparatus (34, 35). Deficiency of Pdcd10 or its ability to bind to STKs impairs STK stability, Golgi assembly, cell orientation, and migration (34–36). Previous studies suggest that PDCD10-STK affects exocytosis of a selective group of vesicles, rather than cause a general defect in secretion (34, 37). This is consistent with our results that PDCD10-STK deficiency only reduces the trafficking and protein level of AQP2 and had no effect on AQP1 and AQP4. Although our in vivo studies suggest that PDCD10 deficiency may not affect AVP-cAMP-PKA–regulated AQP2 trafficking, our in vitro studies suggest that a reduction in PDCD10 could affect both the exocytosis and endocytosis routes of vesicle trafficking. The details regarding molecular and subcellular mechanism warrant further investigation.

Through loss-of-function studies, we revealed the role of PDCD10-STK in regulating Ezrin expression and Aqp2 trafficking and stability, but it is not clear yet what the physiological basis is for the body to evolve this regulatory mechanism. The identification of upstream signaling of PDCD10 and/or mutations or polymorphism in *PDCD10* and *STK* genes associated with human kidney diseases may shed light on their potential roles. Loss of *Krit1* and *Ccm2* in the same lineage of tubular cells did not cause any phenotype. This suggests that the upstream signaling of PDCD10 may lie in the STRIPAK complex, in which PDCD10, STK24, and STK25 are components of the large protein complex, including PP2A and scaffold protein striatin. It has been shown that PDCD10 and STKs as components of STRIPAK complex can promote ERM protein interaction with contractile actomyosin indirectly via PP2A. Depletion of PDCD10, STK24, and MST4 in breast cancer cells promotes the generation of the p-ERM arc in lamellipodia (38). It is possible than PDCD10-STK may function via similar mechanisms to suppress the p-ERM level and permit AQP2 accumulation in the apical membrane.

In the kidneys, Aqp4 localizes to the basolateral membrane of tubular cells and is not subjected to AVP regulation. Interestingly, we found that *Aqp4* is upregulated in both *Pdcd10^KspKO^* and *Stk24/25^dKspKO^* mice that have water reabsorption defects. We hypothesized that an AVP-independent compensatory mechanism is in place to trigger *Aqp4* transcription when water reabsorption is decreased in the *Pdcd10^KspKO^* and *Stk24/25^dKspKO^* mice. It is also possible that excessive Aqp4 protein levels may lead to a defective distribution of intracellular sorting vesicles by occupying more vesicles for basolateral targeting. Indeed, more basolateral translocation of Aqp2 is found in the *Pdcd10^KspKO^* mice after dDAVP treatment when compared with dDAVP-treated littermate controls. In tubular cells, further investigation into the apical versus basolateral vesicle sorting mechanisms would further our understanding of the regulatory mechanisms for Aqp2 apical targeting. Our *Pdcd10^KspKO^* and *Stk24/25^dKspKO^* mice could provide useful models for these studies.

In addition to the kidney, AQP4 is abundantly expressed in the brain, with the highest expression level in the cerebellum (39). AQP4 is polarly expressed in perivascular astrocyte endfeet and mediates bidirectional water transport. It has been shown that AQP4 is involved in astrocyte migration and neural signaling transduction via its regulation of brain extracellular space, and brain edema upon different brain injury scenarios (40, 41). PDCD10 is an causal gene of CCM, a brain vascular disease. CCM has been widely regarded as a disease that originates from defective brain endothelial cells. Loss of function of KRIT1 or CCM2 in endothelial cells, but not neuronal cells, leads to CCM lesion formation (18, 42). However, it has been shown that deletion of Pdcd10 in either endothelial or GFAP-expressing astroglial lineage leads to CCM lesion formation in the brain (43, 44). This suggests a unique role of PDCD10 in CCM pathogenesis. Our finding that PDCD10 could regulate AQP4 expression warrants further investigation regarding whether this is a potential mechanism for the role of PDCD10 in astrocyte in contributing to CCM lesion formation. The high expression level of AQP4 in the cerebellum and high susceptibility of CCM lesion formation in the cerebellum of the CCM mouse model with induced CCM gene deletion is an intriguing association. In addition, both PDCD10 and AQP4 have been shown to be involved in astrocyte activation and migration (44–46). It will be interesting to investigate whether CCM-STK-AQP4 is a critical molecular pathway in regulating the functional communication among endothelial cell–pericyte–astrocyte in neurovascular units.

Our current study identifies a mechanism to regulate AQP2 trafficking and water balance regulation. A clear understanding of this pathway will provide alternative avenues for designing approaches to promote water reabsorption for patients with NDI. Moreover, patients with *PDCD10* mutations develop more aggressive clinical manifestations with greater lesion burden and earlier onset of hemorrhage than patients with CCM caused by *KRIT1* or *CCM2* mutations (47). Whether patients with PDCD10 present defective renal phenotypes has not been studied and would be an area of further investigation.

## Methods

Supplemental methods are available online with this article.

### Mice.

The *Cdh16-Cre* transgenic mice were purchased from The Jackson Laboratory (stock no. 012237). *Krit1^fl/fl^*, *Ccm2^fl/fl^*, and *Pdcd10^fl/fl^* animals have been previously described (18, 48). *Stk24^fl/fl^* and *Stk25^fl/fl^* were generated via ES cell–based gene targeting. Experimental animals were maintained on a 129/C57BL/6J mixed genetic background. All experiments were conducted under the guidelines/regulations of Tianjin Medical University and the guideline of National Research Council (49).

### Metabolic cage studies and urine and plasma analyses.

Sex-matched animals were acclimatized in metabolic cages for 72 hours with free access to water and food before each experiment. The 24-hour urine volume and drinking water consumption were measured on a 72-hour continuous base. For the AVP stimulation test, 1 ng/g body weight of dDAVP (Cayman) was injected i.p. Urine volume and osmolality were measured 6 and 12 hours after treatment. During water deprivation, mice had free access to food, and urine was collected 24 hours after the water bottle was removed. To study the effects of Erlotinib treatment, littermate control and *Pdcd10*-deficient mice received Erlotinib (100 mg/kg/24 hours) (183321-74-6, Cayman) or vehicle only (7.5% ethanol corn oil solution) via oral gavage. Urine samples were harvested from acclimatization day 4 for 3 days before Erlotinib treatment. From the seventh day, mice were treated with Erlotinib or vehicle for 3 days, urine was collected every 24 hours, and volume and osmolarity were measured. At the termination of treatment, animals were euthanized, and kidneys were harvested for Western blotting and IHC analysis as described below.

All collected urine samples were centrifuged at 3000*g* at 4°C for 5 minutes, and the supernatants were saved for use. Osmolality was measured by a freezing-point depression osmometer (SMC 30C-1,Tianjin Tianhe Inc.). Urine urea was measured with the QuantiChrom Urea Assay Kit from Bioassay Systems. Urine biochemistry was analyzed with Cobas 80000-c701 (Roche), and blood biochemistry was analyzed with Beckman Coutler AU5800.

### Cell culture.

The mouse mCCD_Cl1_ cell line (28, 50) was cultured in DMEM/F12 medium (Corning) containing 10 mM HEPES (MilliporeSigma), 2 mM L-glutamine (MilliporeSigma), 100 IU/mL penicillin, 100 IU/mL streptomycin (Invitrogen), 50 nM hydrocortisone (MedChemExpress), 5 pM 3,3,5-triiodo-L-thyronine, (MedChemExpress) 1 nM sodium selenate (Invitrogen), 5 mg/l transferrin (Invitrogen), 10 ng/mL epidermal growth factor (236-EG-200, R&D Systems), and 10% FBS (Biological Industries) at 37°C in a humidified 5% CO_2_ atmosphere. To facilitate the establishment of cell polarity, mCCD_Cl1_ cells were plated on Transwell filters (Costar 3460, Corning) in 12-well plates. Cells form polarized monolayers with 70%–80% confluence after 3 days in culture. For the *Pdcd10* siRNA knockdown assays, mCCD_Cl1_ cells grown on transwell membranes were transfected with siRNA oligonucleotides (MilliporeSigma) using Lipofectamine RNAiMAX Transfection Reagent (Invitrogen) according to the manufacturer’s instruction. To maintain Aqp2 expression, mCCD_Cl1_ cells were infected with Aqp2 lentiviruses with 5 μg/mL polybrene (Solarbio) 24 hours after siRNA transfection. The next day, mCCD_Cl1_ cells were transfected with siRNA for a second time to ensure efficient knockdown of *Pdcd10* gene expression. The cells were treated with 1 nM dDAVP (Cayman) for 4 hours to induce Aqp2 expression maximally.

For Dextran uptake studies, cells were transfected with siRNA and cultured for 72 hours; then, the cells were treated with 2 μM Erlotinib (Cayman) or 10 mM MβCD (MilliporeSigma) for 30 minutes. Dextran-594 (D22913, Invitrogen) was added at a concentration of 0.5 mg/mL for 10 minutes at 37°C. Cells were rinsed 3 times with cold PBS immediately after incubation and fixed with 4% paraformaldehyde (MilliporeSigma).

For immunofluorescence studies, mCCD_Cl1_ cells plated on Transwell filters were fixed with 4% paraformaldehyde for 15 minutes and permeabilized with 0.1% Triton-X/PBS (Solarbio) for 10 minutes. After a 1-hour block with 3% BSA (Solarbio) in PBS, the filters were incubated with the primary antibody overnight at 4°C. The next day, the filters were washed 3 times with PBS and then incubated with the fluorescent tag conjugated secondary antibodies for 1 hour at room temperature. Subsequently, the filters were washed with PBS 3 times and mounted in Vectashield containing DAPI nuclear stain (Vector Laboratories). Immunofluorescence images were obtained using the Zeiss LSM 800 Confocal Microscope, and the fluorescence *z*-slices of the mCCD_Cl1_ cells were generated using ImageJ software (NIH). For all measurements of mean fluorescence intensity (MFI), total fluorescence intensity of an image was recorded in ImageJ and divided by total cell number in the image. The following primary antibodies were used in this study: mouse anti-AQP2 (sc515770; Santa Cruz Biotechnology Inc.; 1:50 dilution), rabbit anti-Rab11a (2413s; Cell Signaling Technology [CST]; 1:50 dilution), and Alexa Fluor 555 Mouse anti-GM130 (560066; BD Biosciences,1:20 dilution).

### Western blotting analysis.

Whole-tissue homogenates were prepared in RIPA buffer (150 mM NaCl, 1%NP-40, 50 mM Tris [pH 8.0]) containing protease and phosphatase inhibitors. Protein concentrations of the homogenates were determined by BCA (Thermo Fisher Scientific, 23252) assay. Aliquots of 30 μg total protein of each sample were separated by SDS-PAGE and transferred to nitrocellulose membrane. The membrane was blocked at room temperature for 1 hour in Tris buffered saline containing 0.1% Tween 20 (Solarbio; PBS-T) containing 5% skim milk and was then incubated with primary antibodies at 4°C overnight. The membrane was washed for 5 minutes with PBS-T buffer 4 times and then incubated with a horseradish peroxide–conjugated secondary antibody at a 1:5000 dilution at room temperature for 1 hour; blots were developed with ECL Western Blotting Substrate (Thermo Fisher Scientific), and the signals were acquired with MiniChemi610 (Beijing Sage Creation Science Co. Ltd) in dark field mode and marker merged mode. The density of the signals acquired at dark filed mode were quantified with ImageJ software. The following primary antibodies were used in this study: anti-AQP1 (ab15080; Abcam; 1:500 dilution), anti-AQP2 (ab199975; Abcam; 1:500 dilution), anti-AQP2(phospho-S256; ab111346; Abcam; 1:100 dilution), anti-AQP4 (ab46182; Abcam; 1:1000 dilution), mouse anti-Ezrin (Developmental Studies Hybridoma Bank [DSHB], 1:500 dilution), phospho-ezrin (Thr567)/radixin (Thr564)/Moesin (Thr558) antibody (3141S; CST; 1:1000 dilution), and anti-ACTB (AC026; ABclonal; 1:50000 dilution).

### Histological analysis.

Kidneys harvested from mice were fixed with 4% PFA and embedded in paraffin. Paraffin sections were stained for H&E and Sirius Red staining using standard protocols. For immunostaining, deparaffinized sections were subjected to rehydration, followed by antigen retrieval by heating in citrate buffer (Beyotime, P0083) for 20 minutes. After blocking in 10% normal donkey serum (Jackson ImmunoResearch) +1% BSA in 0.1% PBS-T for 1 hour, the sections were incubated with primary antibodies overnight at 4°C. The secondary antibodies were incubated for 1 hour. The sections were washed with PBS-T and mounted with mounting solution containing DAPI (Vector Laboratories). The following primary and secondary antibodies were used: rabbit anti-AQP2 (ab199975; Abcam; 1:200 dilution), mouse anti-AQP2 (sc515770; Santa Cruz Biotechnology Inc.; 1:200 dilution), rabbit anti-AQP2(phospho-S256; ab111346; Abcam; 1:200 dilution), goat anti-GFP (ab ab6673; Abcam; 1:200 dilution), phospho-ezrin (Thr567)/radixin (Thr564)/Moesin (Thr558) antibody (3141S; CST; 1:100 dilution), and mouse anti-ezrin (DSHB, 1:100 dilution). Secondary antibody anti-mouse IgG (H+L), F(ab’)2 Fragment (Alexa Fluor 488 conjugate) (4408; CST; 1:500 dilution), anti–mouse IgG (H+L) Alexa Fluor 594 conjugate (8890; CST; 1:500 dilution), anti–rabbit IgG (H+L) Alexa Fluor 594 conjugate (8889; CST; 1:500 dilution), and goat anti–rabbit IgG (H+L) Alexa Fluor 488 conjugate (A11059; Invitrogen; 1:500 dilution) were used. Imaging was performed using a Zeiss Axio-Imager LSM-800 confocal microscope (Carl Zeiss) and Nikon microscope. MFI of images was quantified in ImageJ.

### Real-time PCR analysis.

Total RNA was extracted using Trizol reagents (Thermo Fisher Scientific, 15596018), and complementary DNA (cDNA) was synthesized using StarScript II First-strand cDNA Synthesis Kit (GenStar, A212-10). Real-time PCR was performed with the ChamQ Universal SYBR qPCR Master Mix (Vazyme Biotech Co, Q711-02/03). The following are the primers used in this study: *Aqp1* forward: 5′-AATTCACTTGGCCGCAATGAC-3′; *Aqp1* reverse: 5′-CAGTGGTTTGAGAAGTTGCGG-3′; *Aqp2* forward, 5′-TTACCCCTGTAGAAATCCGCG-3′; *Aqp2* reverse: 5′-AAACCAATGGA GAGAGCAGGG-3′; *Aqp3* forward: 5′-GGTCGACAGAAGGAGTTGATG-3′; *Aqp3* reverse: 5′-GAAGCCAAAAGCCAAGTTGAT-3′; *Aqp4* forward: 5′-TCCAGCTCGATCTTTTGGA CC-3′; *Aqp4* reverse: 5′-CGGGCTTCAGGATCAAGTCTT-3′; *Aqp6* forward: 5′-GCCGTCATTGTTGGGAAGTTC-3′; *Aqp6* reverse: 5′-GGCTCCAGGTCTACCACTTTC-3′; *Aqp7* forward: 5′-TTAGCTTGGTCTGCTGCTTCA-3′; *Aqp7* reverse: 5′-GAACCAAGGC CAAACACCATC-3′; *Aqp11* forward: 5′-GCTTGCTCCTTCTGTAGGTGT-3′; *Aqp11* reverse: 5′-TTGGAGGGTGCTCAGATTGTC-3′; *Nkcc1* forward: 5′-AACACCTACTACCTGCGCAC-3′; *Nkcc1* reverse: 5′-TCCATTCGCAAAGCCATCCT-3′; *Nkcc2* forward: 5′-TTTCAATGGCTTCACTTCTCAGC-3′; *Nkcc2* reverse: 5′-CGAACTTGACGGTAACTCTT-3′; *α-Enac* forward: 5′-GAGGAATACTTCAGCTACCCCG-3′; *α-Enac* reverse: 5′-AAAAAGCGTCTGTTCCGTGATG-3′; *β-Enac* forward: 5′-TTCCAGTACTCCAAGGTCAAGC-3′; *β-Enac* reverse: 5′-TCCGCTCATCAATAAGGACCAG-3′; *γ-Enac* forward: 5′-TGAGAACGAGAAGGGAAAGGC-3′; *γ-Enac* reverse: 5′-TTTGTACCACTCCTGGATGGC-3′; *Cldn2* forward: 5′-TTCCAGAGCTCTTCGAAAGGAC-3′; *Cldn2* reverse: 5′-TATCTTCGGAGCCTGTTTGCTT-3′; *Ezrin* forward: 5′-CGCCTGAGAATTAACAAGCGG-3′; *Ezrin* reverse: 5′-CAGTTGGAGGGCCTTCTCAAT-3′; *Radixin* forward: 5′-AGTTGGCTTACGTGAGGTCTG-3′; *Radixin* reverse: 5′-ACACGCTGTGGTAAGAGTCTG-3′; *Moesin* forward: 5′-TGCACAAGTCTGGCTACCTG-3′; *Moesin* reverse: 5′-CTCCACGGGAAGCCAATCTT-3′; *Avpr2* forward, 5′-CCGGGAGATACATGCCAGTC-3′; and *Avpr2* reverse: 5′-GCATGAGCAACACAAAGGGG-3′.

### Statistics.

All the statistical analysis in the study was done using 2-tailed *t* tests and 1-way ANOVA in GraphPad Prism statistical software. Values are presented as mean ± SEM. Statistical significance was considered when *P* ≤ 0.05.

### Study approval.

The Institutional Animal Care and Use Committee (IACUC) of Tianjin Medical University approved all animal ethics and protocols.

## Author contributions

RW and STW designed and performed most of the experiments and wrote the manuscript. XY, YQ, JPC, RG, SS, YW, TZ, and ZH performed experiments and helped with data analysis. JJLW, HAL, and SIA helped with data analysis. RL helped with data analysis and writing the manuscript. YZ and YX provided critical reagents and performed experiments. XZ designed experiments and wrote the manuscript.

## Supplementary Material

Supplemental data

## Version 1 06/22/2021

Electronic publication
