# Supplementary material for: Pdcd10-Stk24/25 complex controls kidney water reabsorption by regulating Aqp2 membrane targeting
Source: JCI Insight. 2021 Jun 22;6(12):e142838. doi: 10.1172/jci.insight.142838 (PMC8262504; doi:10.1172/jci.insight.142838)
Supplement: Supplemental data [file jciinsight-6-142838-s135.pdf]

Supplemental Information

Supplemental Figure 1-13

Supplemental Materials and Methods

Supplemental Figures

Supplemental Figure 1

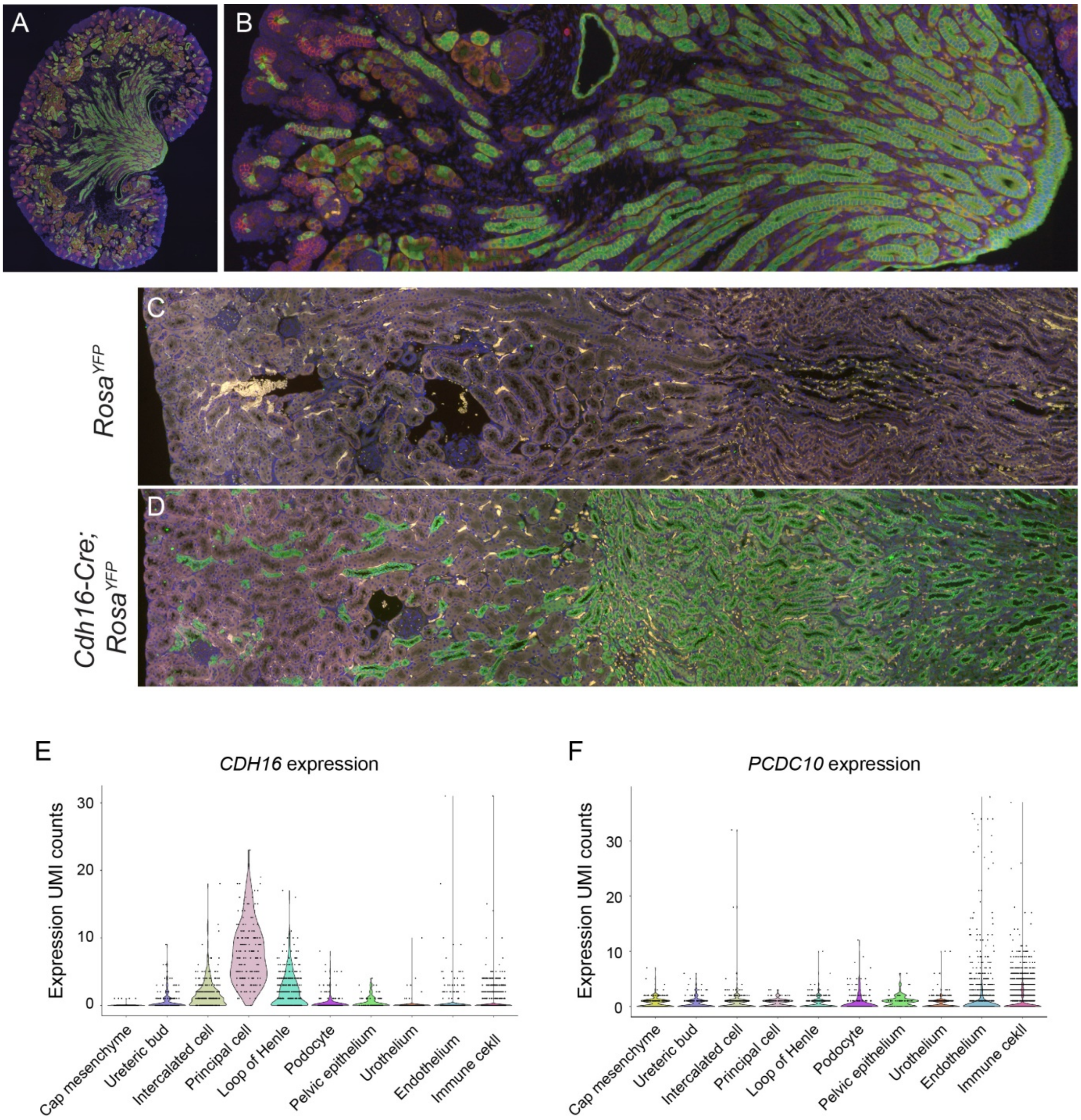

Fig. S1. *Cdh16cre* lineage tracing and single cell RNA sequencing analysis of *CDH16* and *PDCD10* expression in kidneys. (**A, B**) Immunofluorescence stainings of GFP (green), E-Cadherin (red) and DAPI (blue) on sections of kidneys from *Cdh16cre*;*Rosa*^{YFP} at P1 of age. (**C, D**) Immunofluorescence assays of GFP (green), E-Cadherin (white) and DAPI (blue) on

sections of kidneys from control (*Rosa*^{YFP}) (**C**) and lineage labeled (*Cdh16cre*;*Rosa*^{YFP}) mice (**D**) at 13 weeks of age. (**E, F**) Violin plots showing the expression patterns of *CDH16* and *PDCD10* in the human kidney from single cell RNA-seq database.

Supplemental Figure 2

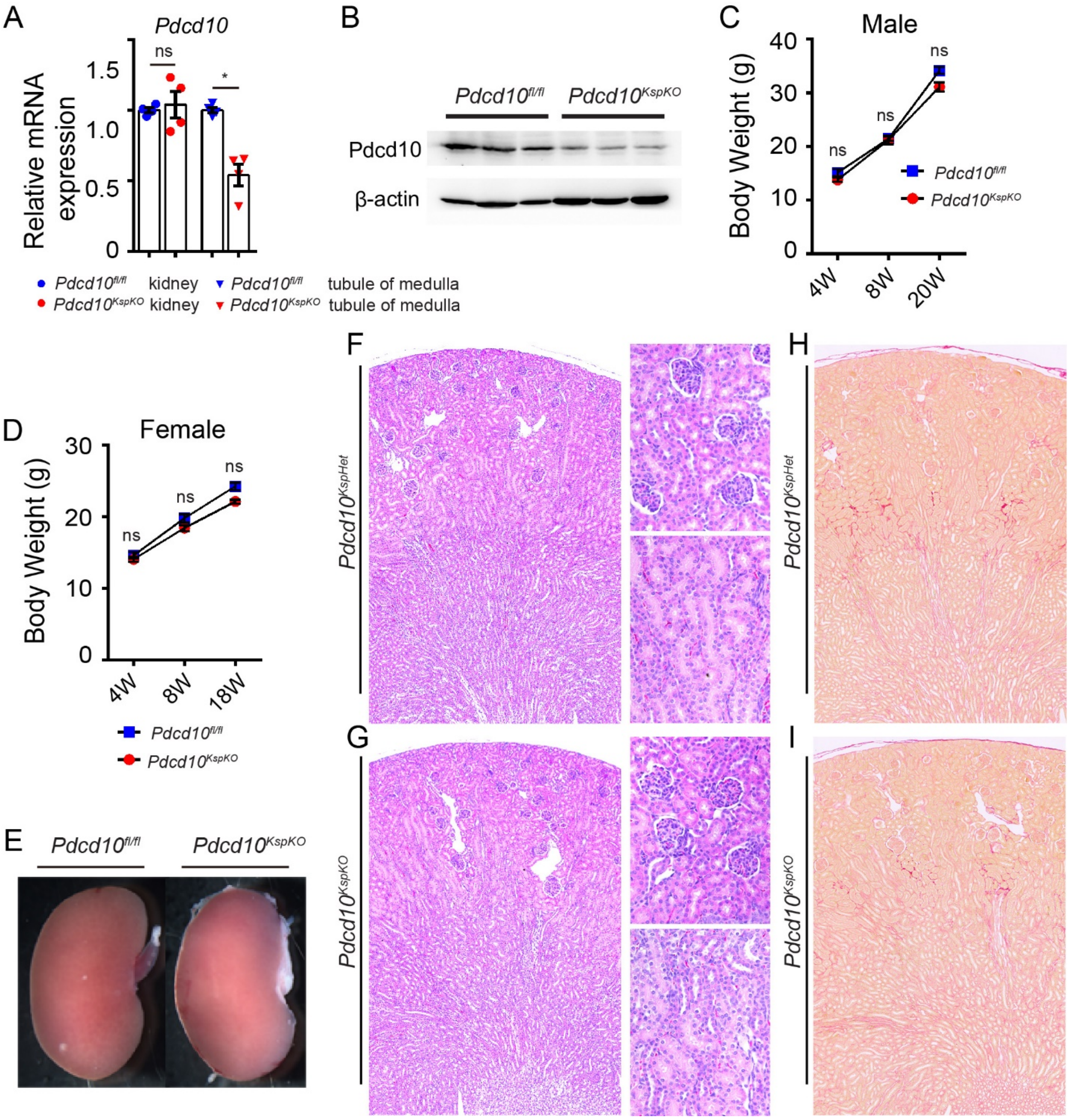

Fig. S2. No gross histological defect is detected in the *Pdcd10*-deficient mice.

(**A**) The relative expression level of *Pdcd10* demonstrates ~50% decreased expression in *Pdcd10* deficient mouse kidney tubules, but no significantly change for the samples from whole kidney tissues (n =4 for each group). (**B**) Western blots of Pdcd10 protein demonstrate the downregulation of Pdcd10 in the kidney medulla of *Pdcd10*^{KspKO} mice at 4 weeks of age (n=3).

(**C, D**) Growth plots show no significant difference in body weight between control and *Pdcd10* deficient mice from 4-20 weeks of age (male *Pdcd10*$^{fl/fl}$, n =6; male *Pdcd10*^{KspKO}, n =9; female *Pdcd10*$^{fl/fl}$, n =8; male *Pdcd10*^{KspKO}, n =11). (**E**) Images show no gross difference between control and *Pdcd10* deficient kidneys at 4 weeks of age. (**F, G**) Representative images of haematoxylin and eosin (HE) staining of control (**F**) and *Pdcd10* deficient mice (**G**), higher magnification views of renal cortex with glomeruli and medulla with tubules show no overt histological defect in *Pdcd10* deficient kidney. (**H, I**) Representative images of Sirius Red staining on kidney sections of control (**H**) and *Pdcd10* deficient mice (**I**) show no fibrosis damage. Data in quantitative plots are presented as mean ± SEM using the unpaired t-test. **P<0.01; ns: not significant.

Supplemental Figure 3

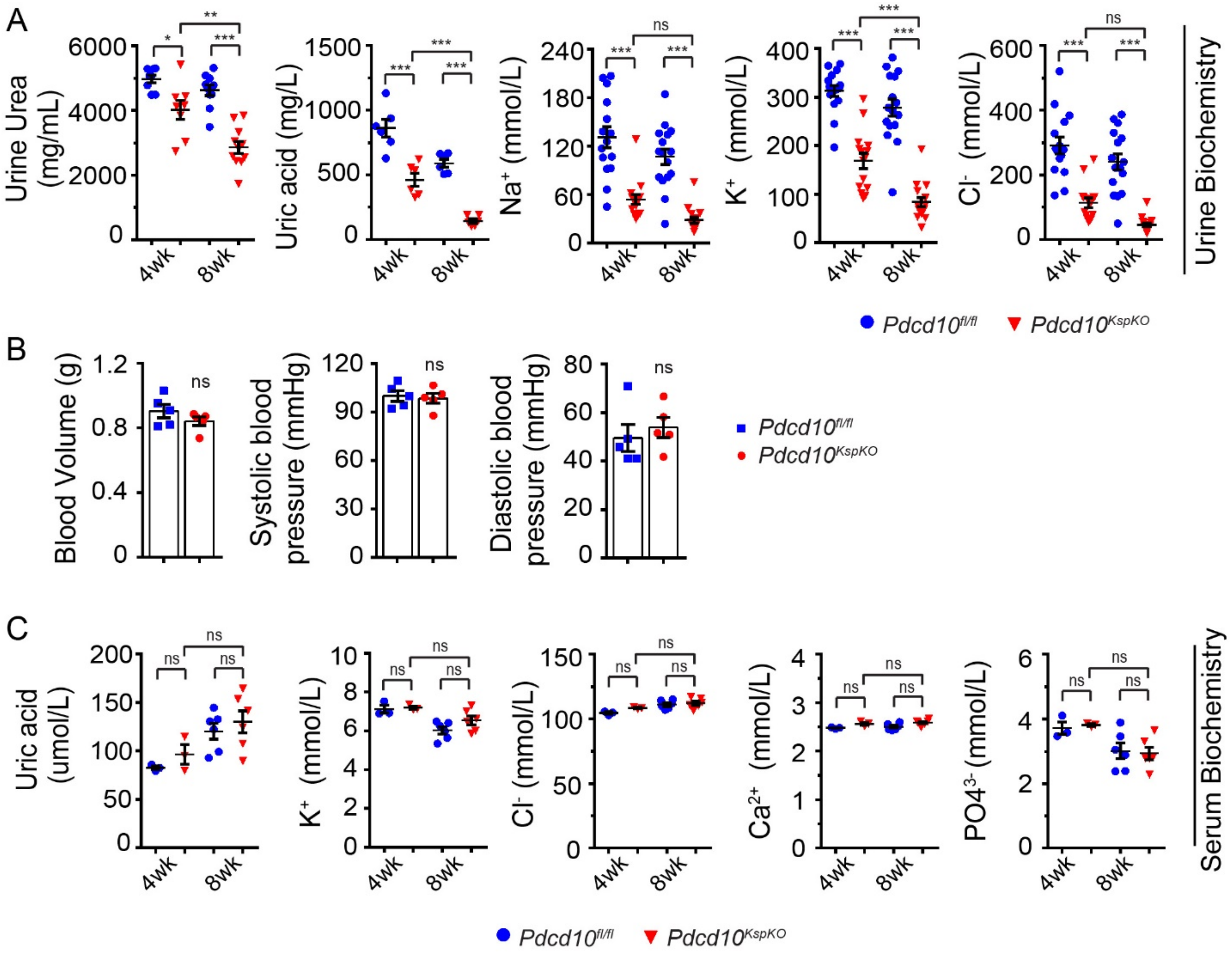

Fig. S3. Urine and serum biochemistry of control and *Pdcd10*-deficient mice at 4 and 8 weeks of age. (**A**) The concentration of urine urea, uric acid, sodium, potassium, chloride is decreased in *Pdcd10* deficient mice at both 4 and 8 weeks of age (n = 6-15 for 4wk mice, n = 6-16 for 8wk mice). (**B**) No significant difference was detected for blood volume, systolic blood pressure, diastolic blood pressure between control and *Pdcd10* deficient mice at 10wk of age (n=5 for each group). (**C**) Serum concentration of uric acid, potassium, chloride, calcium, and phosphate remain unchanged between control and *Pdcd10* deficient mice at 4 and 8 weeks of age (n =3 for 4wk mice, n =6 for 8wk mice). Data in quantitative plots are presented as mean ± SEM using the unpaired t-test or 1-way ANOVA. *P<0.05; **P<0.01; ***P<0.001; ns: not significant.

Supplemental Figure 4

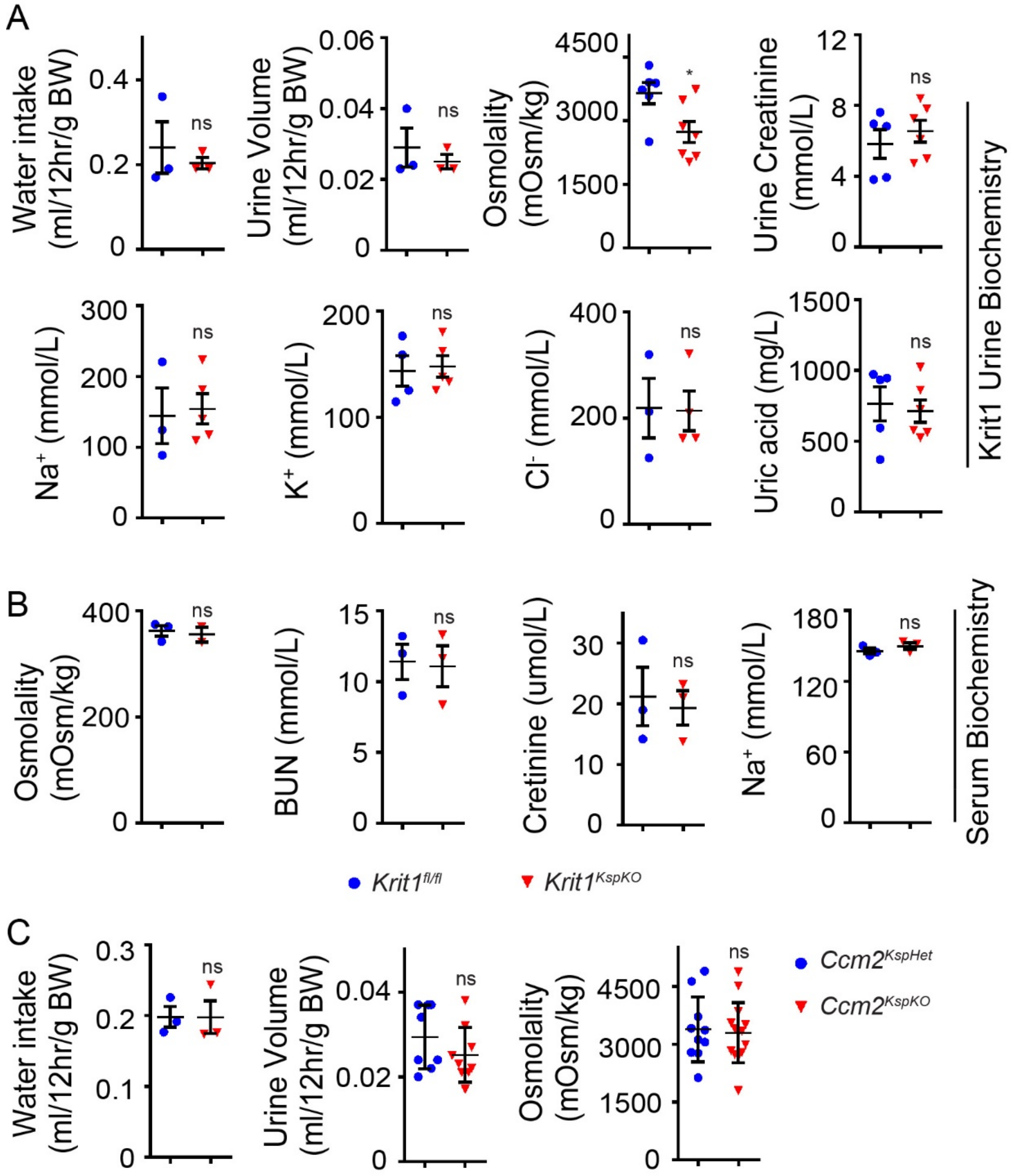

Fig. S4. Deletion of *Krit1* or *Ccm2* in kidney tubules does not cause polyuria. (**A**) No significant difference is detected for water intake, urine volume, urine osmolality, urine concentration of creatinine, sodium, potassium, chloride, uric acid between control and *Krit1* deficient mice at 20 weeks of age (n = 3-6 for *Krit1*$^{fl/fl}$ mice, n = 3-7 for *Krit1*^{KspKO} mice). (**B**) Plasma osmolality, blood urea nitrogen, the concentration of creatinine and sodium are not significantly different between control and *Krit1* deficient mice (n = 3). (**C**) No significant

difference is detected for water intake, urine volume, and urine osmolality between control and *Ccm2*-deficient mice at 24 weeks of age (n = 3-10 for $Ccm2^{KspHet}$ mice, n = 3-14 for $Ccm2^{KspKO}$ mice). Data in quantitative plots are presented as mean ± SEM using the unpaired t-test. *P<0.05; ns: not significant.

Supplemental Figure 5

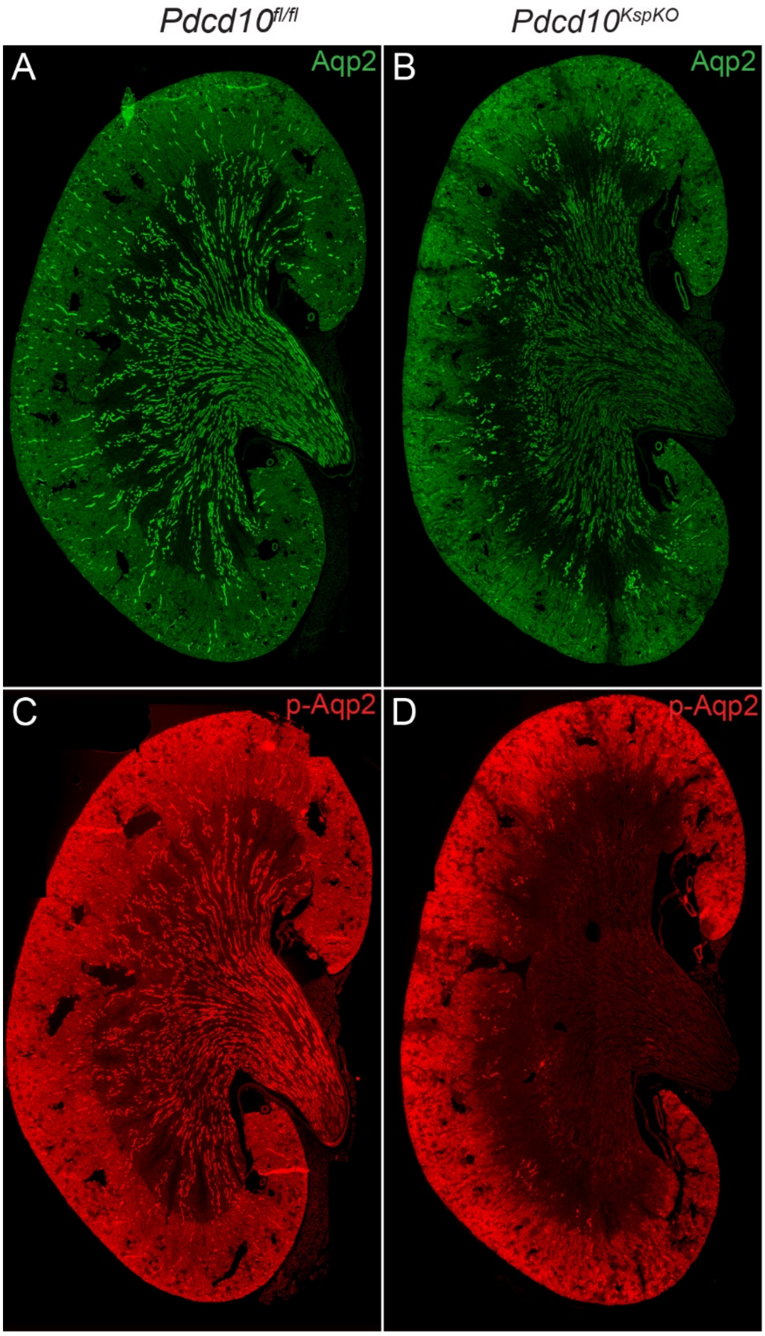

Fig. S5. *Pdcd10* deficiency leads to decreased expression of Aqp2 and p-Aqp2 in the renal medulla. (**A, B**) Immunostaining images of Aqp2 on kidney sections from control (**A**) and *Pdcd10*-deficient mice (**B**) at 4 weeks of age. (**C, D**) Immunostaining images of pS256-Aqp2 on sections from control (**C**) and *Pdcd10*-deficient kidneys (**D**) at 4 weeks of age. Note the diffused fluorescence signal in the cortex are non-specific stainings.

Supplemental Figure 6

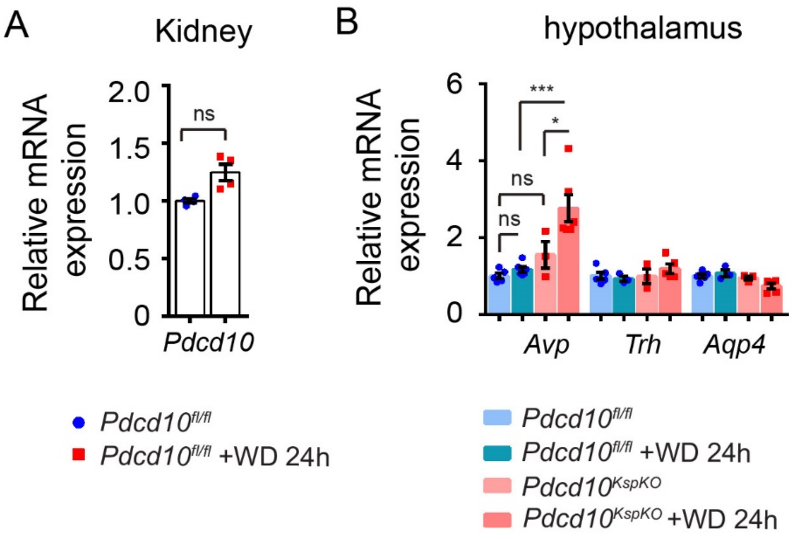

Fig. S6. Expression of *Pdcd10* in kidney and Avp in hypothalamus upon water deprivation. A) Relative expression level of *Pdcd10* in the kidney of control mice before and 24 hours after water deprivation (n = 4 for each group). (**B)** Relative expression level of *Avp*, *Trh*, *Aqp4* in the hypothalamus of control and *Pdcd10*-deficient mice before and 24 hours after water deprivation (n = 4 for each group). Data are presented as mean ± SEM. Asterisks indicate significance relative to the control group determined by the unpaired t-test or 1-way ANOVA. *P<0.05; ***P<0.001; ns: not significant.

Supplemental Figure 7

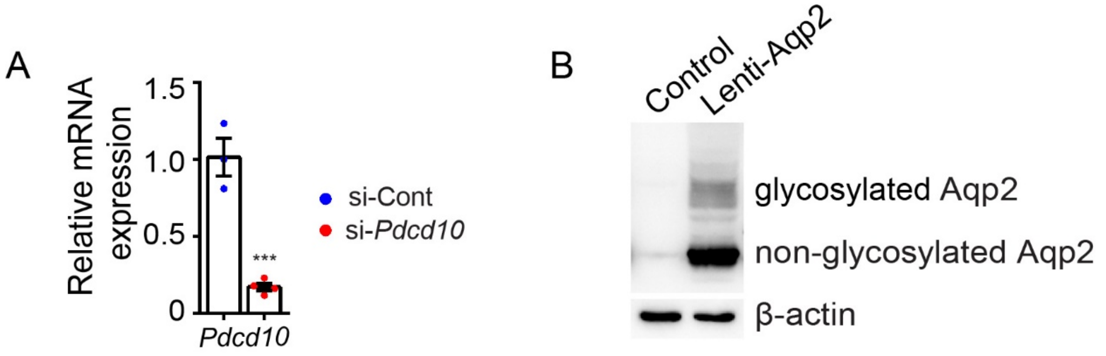

Fig. S7. The efficiency of *Pdcd10* knockdown and the expression of Aqp2 *in vitro*. (**A**) The relative expression level of *Pdcd10* demonstrates ~75% decreased in si-*Pdcd10* treated mCCD$_{Cl1}$ cells. (si-Cont, n = 3; si-*Pdcd10*, n = 4). (**B)** Immunoblots demonstrates efficient Aqp2 expression in cells transduced with Aqp2 expressing lentivirus. Data in quantitative plots are presented as mean ± SEM using the unpaired t-test. ***P<0.001.

Supplemental Figure 8

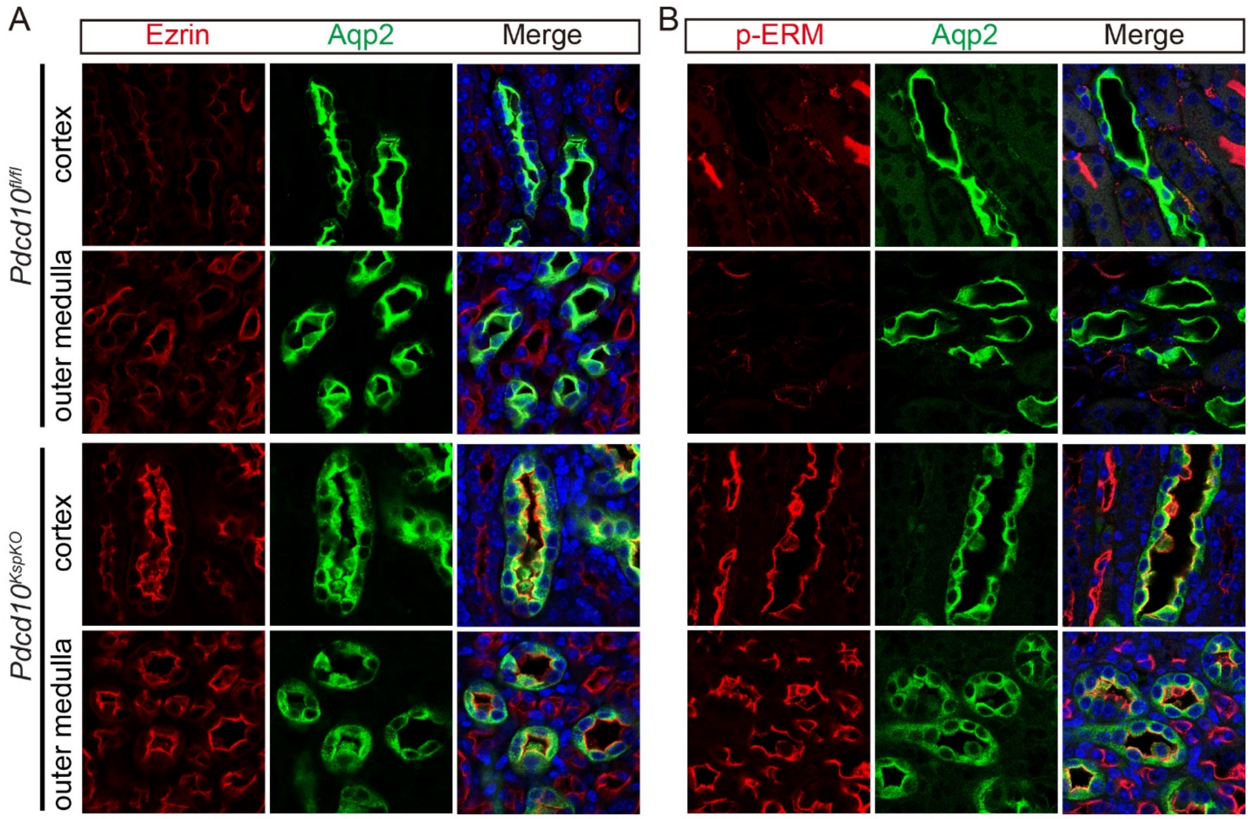

Fig. S8. Deletion of *Pdcd10* increased Ezrin and p-ERM in distal tubules of the renal cortex and outer medulla. (**A**) Immunofluorescence stainings of Ezrin (red) and Aqp2 (green) in the kidneys of control and *Pdcd10* deficient mice show the increased expression and localization of Ezrin on the luminal membrane of tubules in *Pdcd10* deficient mice. (**B**) Immunofluorescence stainings of p-ERM (red) and Aqp2 (green) in the kidneys of control and *Pdcd10* deficient mice show the increased expression and luminal membrane localization of p-ERM in *Pdcd10* deficient mice.

Supplemental Figure 9

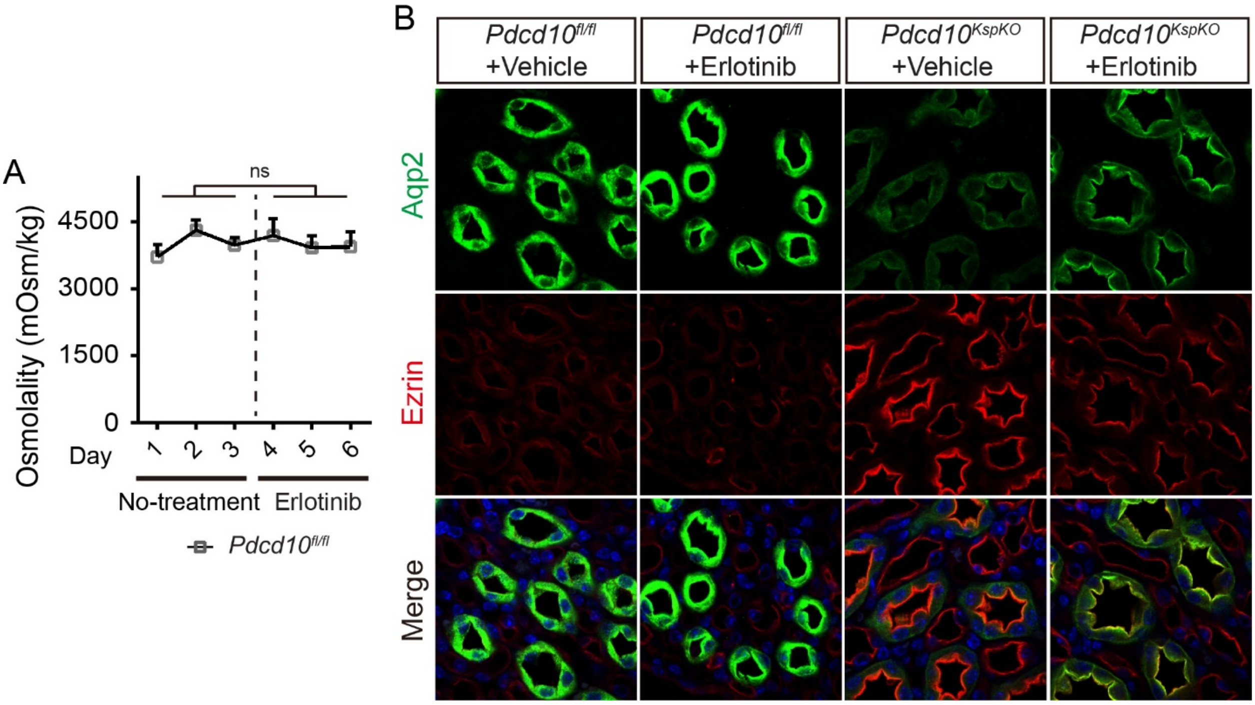

Fig. S9. Erlotinib treatment increases Aqp2 abundance in the apical membrane. (A) Erlotinib treatment do not significantly alter urine osmolarity in control mice (n = 7). **(B)** Immunofluorescence staining of Aqp2 (green) and Ezrin (red) on sections of kidney medulla of control and Pdcd10-deficient mice show the increased p-Aqp2 expression in correlation with the decrease of Ezrin expression after administration of Erlotinib for 3 days. Data in quantitative plots are presented as mean ± SEM using the unpaired t-test. ns: not significant.

Supplemental Figure 10

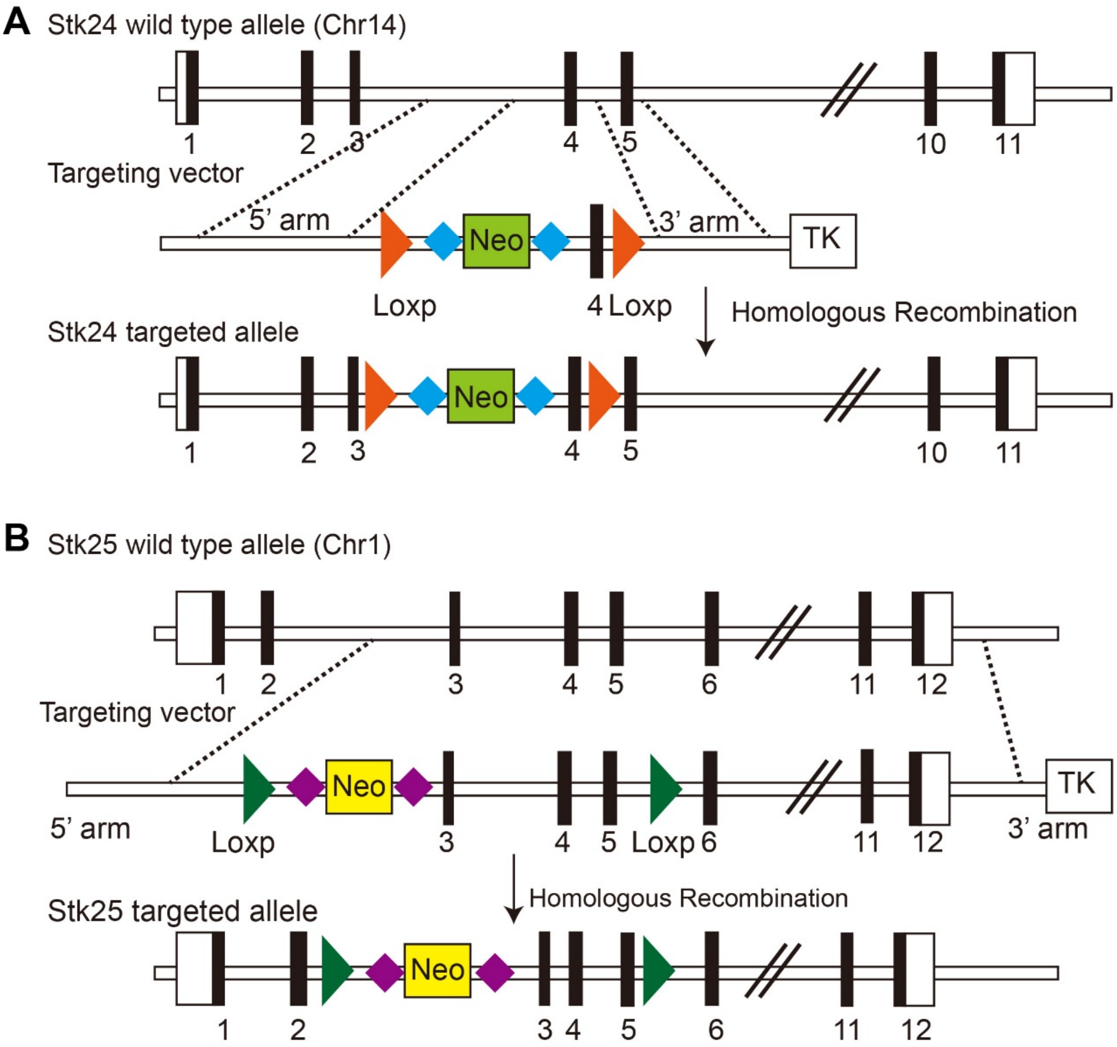

Fig. S10. Schematic of the construction of *Stk24*$^{fl/fl}$ and *Stk25*$^{fl/fl}$ targeting allele. (A) Stk24 gene targeting strategy. LoxP sites were inserted to upstream and downstream of exon 4 of *Stk24* gene for conditional deletion of Stk24 expression. (B) Stk25 gene targeting strategy. LoxP sites were inserted to upstream of exon 3 and downstream of exon 5 of *Stk25* gene for conditional deletion of Stk25 expression.

Supplemental Figure 11

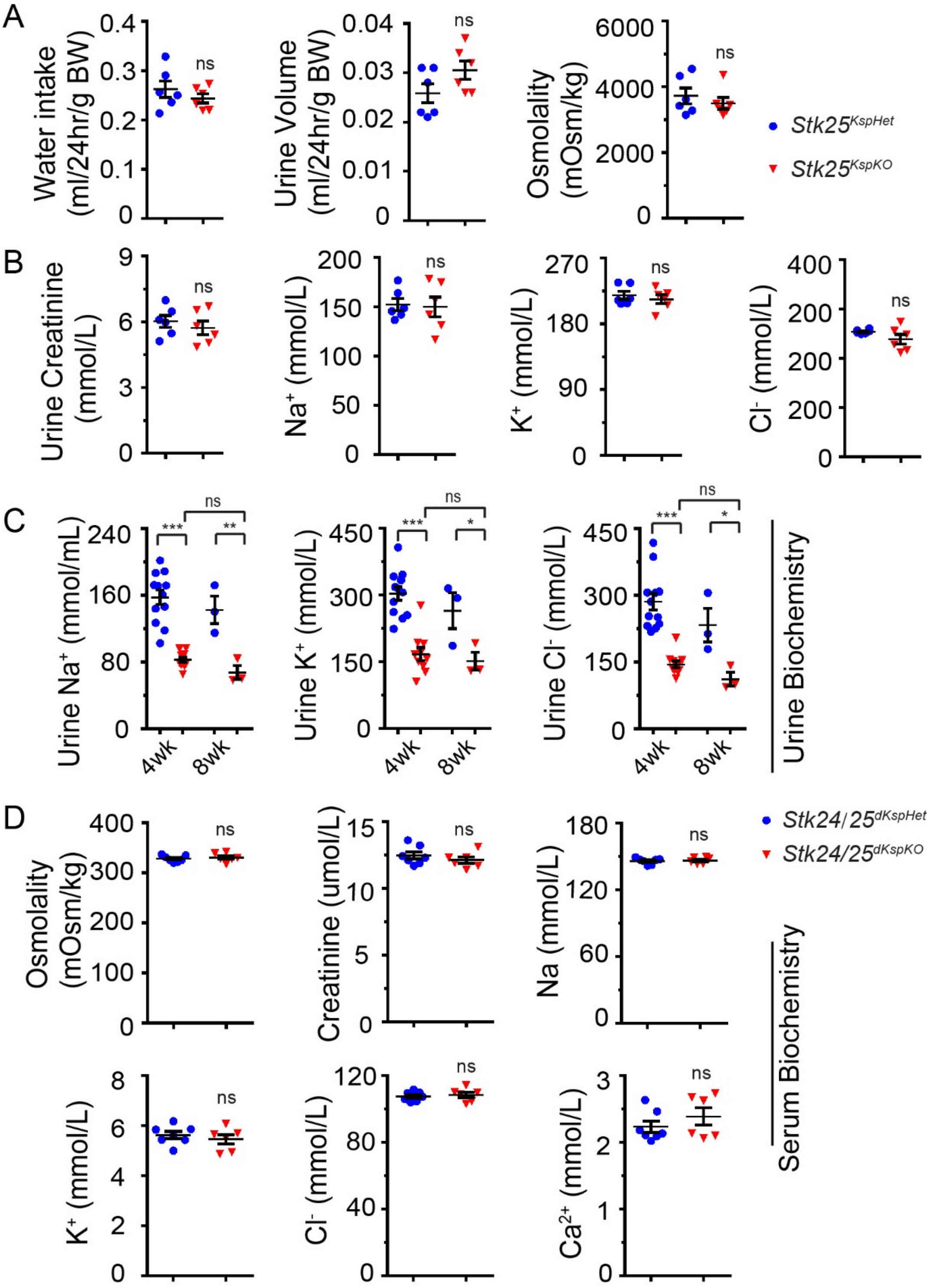

Fig. S11. Biochemistry of renal function in the *Stk25*^{KspKO} and *Stk24/25*^{dKspKO} mice. (**A, B**) No significant difference is detected for water intake, urine volume, urine osmolality, the concentration of creatinine, sodium, potassium and chloride in the urine from control and *Stk25*

deficient mice at 8 weeks of age (n = 6 for each group). (**C**) Urine concentration of sodium, potassium, and chloride are decreased in *Stk24*/*Stk25* deficient mice at 4 and 8 weeks of age (n = 10-12 for 4wk mice, n = 3 for 8wk mice). (**D**) Plasma osmolality, and the concentration of creatinine, sodium, potassium, chloride and calcium are unchanged between control and *Stk24*/*Stk25* deficient mice (n = 7 for *Stk24/25*$^{fl/fl}$ mice, n = 6 for *Stk24/25*^{dKspKO} mice). Data in quantitative plots are presented as mean ± SEM using the unpaired t-test or 1-way ANOVA. *P<0.05; **P<0.01; ***P<0.001; ns: not significant.

Supplemental Figure 12

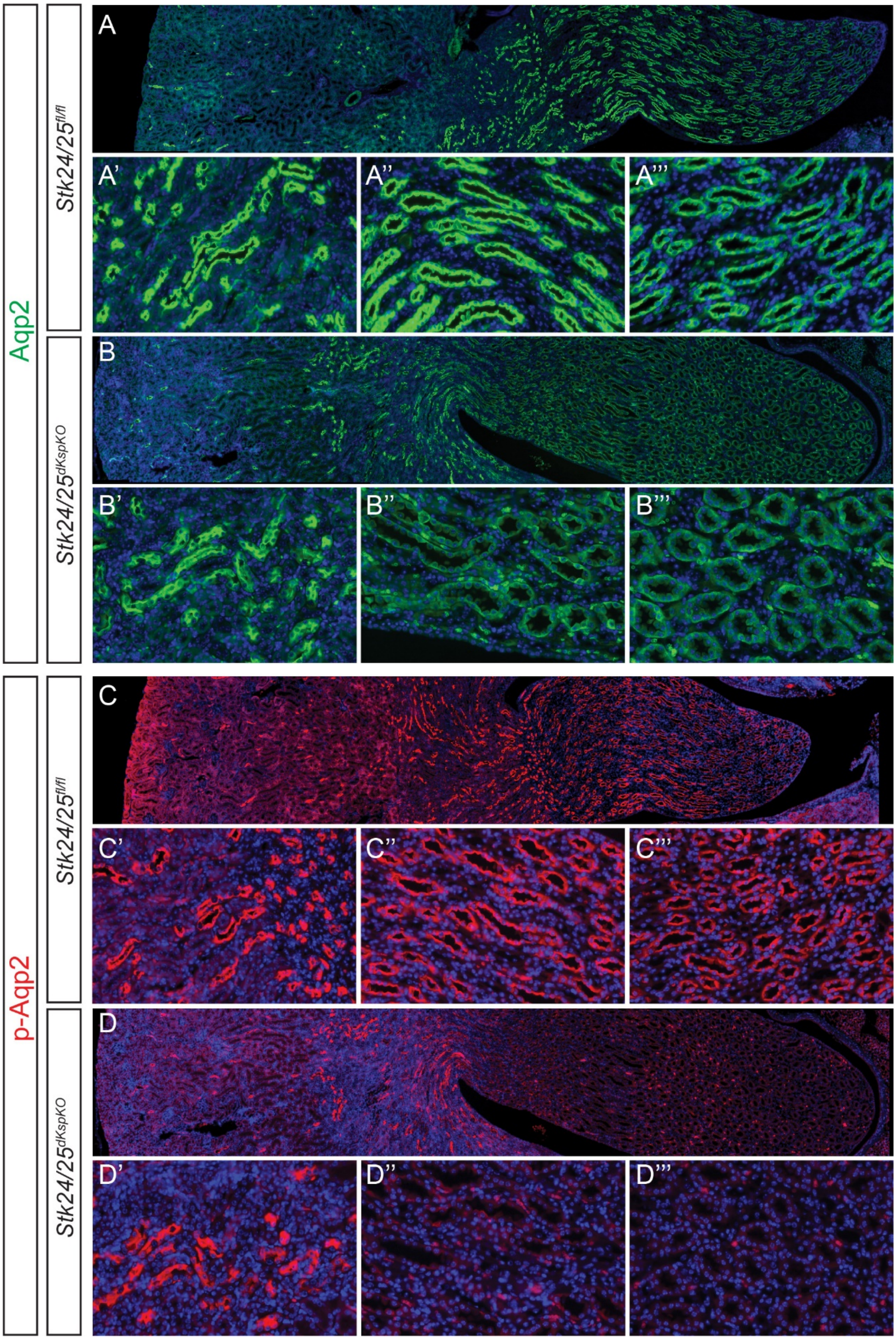

Fig. S12. Double deletion of *Stk24 and Stk25* in the kidneys decrease Aqp2 and pS256-Aqp2 protein levels. (**A, B**) Immunostainings of Aqp2 on kidney sections of control (**A**) and the *Stk24/25*^{dKspKO} kidneys **(B)** with higher magnification view of the outer medulla (**A', B'**), inner medulla (**A'', B''**) and renal papilla (**A''', B'''**). **(C, D)** Immunostaining of pS256-Aqp2 in the control **(C)** and *Stk24/25*^{dKspKO} **(D)** kidneys, with higher magnification views of the outer medulla (**C', D'**), inner medulla (**C'', D''**), and renal papilla (**C''', D'''**).

Supplemental Figure 13

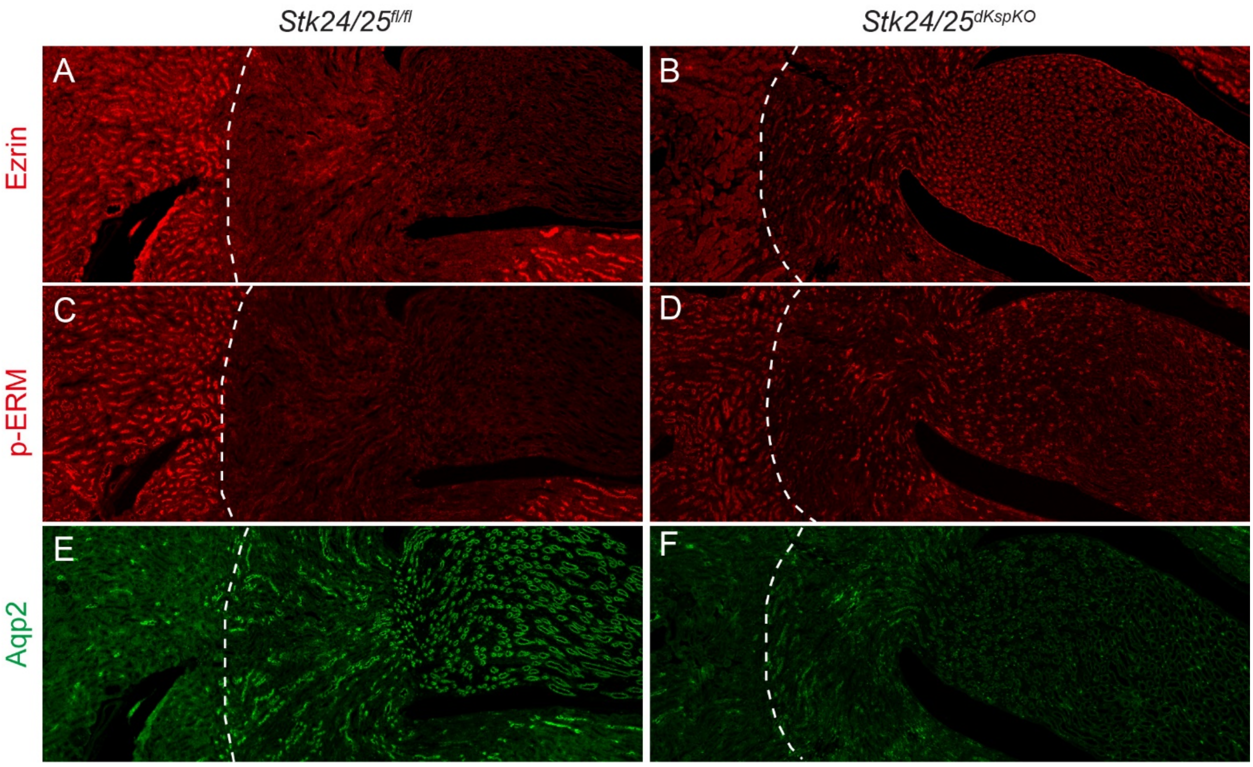

Fig. S13. Deletion of *Stk24 and Stk25* increases Ezrin and p-ERM expression in kidney medulla. Immunofluorescence stainings of Ezrin (**A, B**), p-ERM (**C, D**) demonstrate the protein level of both Ezrin and p-ERM are increased in the medulla of *Stk24/Stk25* deficient kidneys, and correlated to the decreased expression of Aqp2 (**E, F**).

Supplemental Materials and Methods

Primary antibodies used in the supplemental study

anti-Pdcd10 (10291-2-AP; Proteintech; 1:300 dilution for western blotting analysis)

qPCR primers used in the supplemental study

Avp forward: 5'-TCTCCTCCGCCTGCTACTT-3'

Avp reverse: 5'- GCGAGGGCAGGTAGTTCTC-3'

Trh forward, 5'- TCTGGCTTTGATCTTCGTGCT -3'

Trh reverse: 5'-GTCTTCCTCCTTCTCCTCCCT -3'

Single cell RNA-seq analysis

To analyze the gene expression of CDH16 and PDCD10 in human kidney, we made use of the human kidney single cell RNAseq data from previous published study (PMID: **30093597**). The raw reads count data and cell type annotation information were extracted from the provided data. The total reads counts were normalized to 10,000 for each cell. Then the normalized values for CDH16 and PDCD10 in each cell type were visualized in ggplot2 package (version 3.2.1) in R software (version 3.6.1).

Microdissection of mouse renal medulla tubules

Mice were anesthetized with avertin and perfused with PBS. The kidneys were quickly removed, 0.5~1 mm tissue slices were cut from the middle part of the kidney, and medulla and cortex tissue were separated with surgical knife. The medulla tissues were transferred to a solution containing 2 g/L collagenase IV (Sigma) and digested for 25 min at 37°C with gentle agitation. The renal tubules were recovered under a stereomicroscope in cold PBS buffer after incubation.

Blood volume and blood pressure measurements

10-12 weeks old female mice were used for blood volume and blood pressure measurements. For blood volume measurement, animals were euthanized with CO_2 and trunk blood was collected. Then blood volume was quantified by weight. Blood pressure was

measured using a Visitech BP-2000. In brief, systolic blood pressure (SBP) and diastolic blood pressure (DBP) was determined by eighteen consecutive measurements made under stable heart rate condition using the tail cuff method, and each mouse was continuously measured for 3 days to get effective mean SBP and DBP.
